# Genetic signature of human longevity in PKC and NF‐κB signaling

**DOI:** 10.1111/acel.13362

**Published:** 2021-07-01

**Authors:** Seungjin Ryu, Jeehae Han, Trina M. Norden‐Krichmar, Quanwei Zhang, Seunggeun Lee, Zhengdong Zhang, Gil Atzmon, Laura J. Niedernhofer, Paul D. Robbins, Nir Barzilai, Nicholas J. Schork, Yousin Suh

**Affiliations:** ^1^ Department of Genetics Albert Einstein College of Medicine Bronx NY USA; ^2^ The Scripps Research Institute La Jolla CA USA; ^3^ Department of Biostatistics University of Michigan Ann Arbor MI USA; ^4^ Department of Medicine Albert Einstein College of Medicine Bronx NY USA; ^5^ Department of Biology Faculty of Natural Sciences University of Haifa Haifa Israel; ^6^ Insitute on the Biology of Aging and Metabolism University of Minnesota Minneapolis MN USA; ^7^ J. Craig Venter Institute La Jolla CA USA; ^8^ Department of Ophthalmology and Visual Sciences Albert Einstein College of Medicine Bronx NY USA; ^9^ Departments of Obstetrics and Gynecology, and Genetics and Development Columbia University New York NY USA; ^10^ Present address: Department of Comparative Medicine and Immunobiology Yale University New Haven CT USA; ^11^ Present address: Department of Epidemiology University of California Irvine CA USA

**Keywords:** centenarian, genetic variant, longevity, NF‐κB, PKC, rare variant

## Abstract

Gene variants associated with longevity are also associated with protection against cognitive decline, dementia and Alzheimer's disease, suggesting that common physiologic pathways act at the interface of longevity and cognitive function. To test the hypothesis that variants in genes implicated in cognitive function may promote exceptional longevity, we performed a comprehensive 3‐stage study to identify functional longevity‐associated variants in ~700 candidate genes in up to 450 centenarians and 500 controls by target capture sequencing analysis. We found an enrichment of longevity‐associated genes in the nPKC and NF‐κB signaling pathways by gene‐based association analyses. Functional analysis of the top three gene variants (*NFKBIA*, *CLU*, *PRKCH*) suggests that non‐coding variants modulate the expression of cognate genes, thereby reducing signaling through the nPKC and NF‐κB. This matches genetic studies in multiple model organisms, suggesting that the evolutionary conservation of reduced PKC and NF‐κB signaling pathways in exceptional longevity may include humans.

## Graphical abstract

To test the hypothesis that variants in genes implicated in cognitive function may promote exceptional longevity, we performed a 3‐stage study to identify functional longevity‐associated variants in ~700 candidate genes in up to 450 centenarians and 500 controls by target capture sequencing analysis. We found an enrichment of longevity‐associated genes in the nPKC and NF‐kB signaling pathways by gene‐based association analyses. Functional analysis of the top three gene variants (NFKBIA, CLU, PRKCH) suggests that non‐coding variants reduce signaling through the nPKC and NF‐kB.

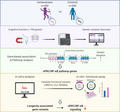

## 1 INTRODUCTION

The heritability of life expectancy is estimated to be ~25% (Herskind et al., 1996; McGue et al., 1993), to lower than 10% considering inherited sociocultural factors (Ruby et al., 2018) in the general population, but it becomes more substantial after age 65 and 85 years, at 36% and 40% (Murabito et al., 2012), respectively. Family studies suggest that the genetic component of life expectancy is especially strong in the oldest old such as centenarians, who live 100 years or more (Adams et al., 2008; Barzilai et al., 2001). These studies support the utility of centenarians as a human model system of exceptional longevity, “decelerated” aging or “healthy” agers.

Being a centenarian is rare (only 1 in 5000 people lives to 100 years in the United States) (Andersen et al., 2012) despite the recent increase in the life expectancy of the general population. In addition to an extended lifespan, centenarians have an extended healthspan via delaying, surviving, or escaping age‐associated diseases including cardiovascular diseases, cancer, and neurodegenerative diseases (Ailshire et al., 2015; Hitt et al., 1999). Thus, exceptional longevity is obviously coupled with exceptional resistance to diseases that lead to earlier mortalities in humans.

Individuals with exceptional longevity manifest delayed onset of Alzheimer's disease (AD) and dementia (Kliegel et al., 2004; Perls, 2004). Genetic studies indicate that longevity‐associated genes may be protective against cognitive decline, dementia, and AD (Barzilai et al., 2006; Christensen et al., 2006; Dubal et al., 2014; Sanders et al., 2010; Sebastiani et al., 2012). The *APOE* gene, encoding apolipoprotein E, is a prime example shown to be associated with both AD and longevity (Christensen et al., 2006). Centenarians are depleted of AD‐predisposing *APOE* ε4 allele, while they are enriched with the AD‐protective *APOE* ε2 allele. A functional longevity‐associated allele in the cholesteryl ester transfer protein (*CETP*) gene, I405V, is also significantly associated with slower memory decline and lower risk for dementia and AD (Barzilai et al., 2006; Sanders et al., 2010). Longevity‐associated KL‐VS variants (haplotype including F352V and C370S) in the Klotho gene (*KLOTHO*) are also associated with protection against cognitive decline (Dubal et al., 2014). A genome‐wide association study (GWAS) revealed that variants associated with longevity are most significantly enriched in genes related to AD and dementia (Sebastiani et al., 2012). These results suggest that there are common underlying pathways between longevity and resilience to AD and related cognitive decline. In addition, as shown in the *APOE* gene, different alleles in the same gene can be associated with opposing outcomes to either accelerate cognitive decline and increase neurodegenerative disease risk or promote longevity and healthy aging in the brain. A rare variant (A673T) discovered in the well‐known familial AD causal gene, amyloid precursor protein (*APP*), protects against AD, and related cognitive decline (Jonsson et al., 2012). Taken together, genes associated with cognitive function have emerged as interesting candidate genes that might contribute to human longevity.

**FIGURE 1:**
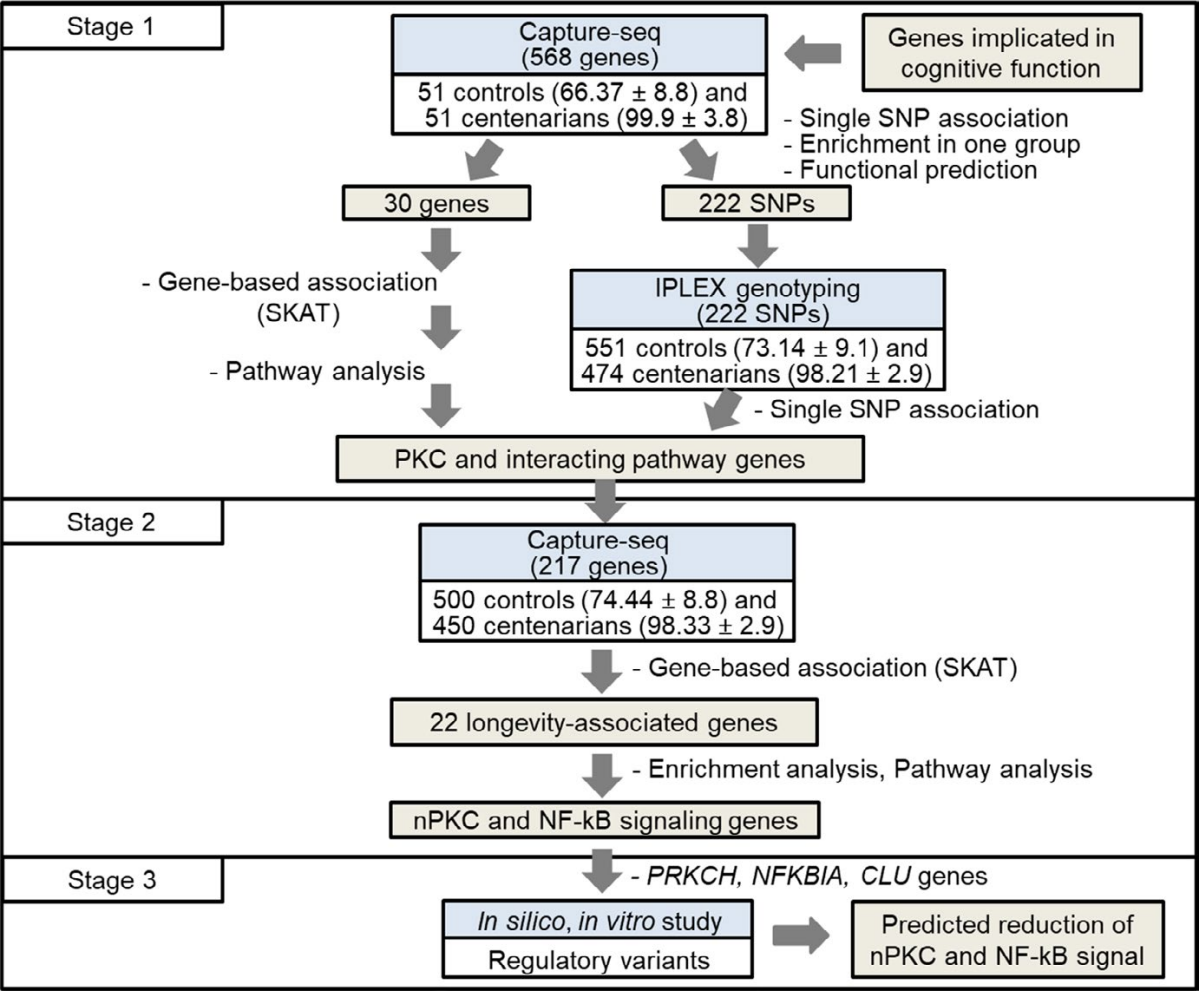
Workflow of genetic study to discover longevity‐associated genes with functional variants in candidate genes. A 3‐stage study design was used for genetic discovery. In Stage 1 with 568 genes identified candidate genes in PKC and interacting pathways for the stage 2. The stage 2 study included 217 genes and identified 22 longevity‐associated genes by Captures‐seq with larger population. In Stage 3, the top functionally important pathways enriched with longevity‐associated genes were selected. The impact of non‐coding variants was studied *in silico* and *in vitro* to predict the impact on gene expression and therefore the impact on signaling. Mean with standard deviation of age of each group was indicated

In this study, we tested the hypothesis that variants in genes implicated in cognitive function may promote exceptional longevity in humans. We performed a comprehensive 3‐stage study to identify functional longevity‐associated variants in a total of 701 candidate genes by capture sequencing analysis in up to 450 long‐lived individuals (95 years of age or older; defined as centenarians in this study) and 500 controls of Ashkenazi Jewish descent followed by functional studies to ascertain the biological significance of the variant (Figure 1). Here, we report an enrichment of longevity‐associated genes in the novel PKC (nPKC) and NF‐κB signaling pathways among our candidate genes. Functional analysis of top 3 longevity‐associated gene variants (*PRKCH*, *NFKBIA*, *CLU*) *in vitro* suggests that non‐coding variants modulate the expression of cognate genes, thereby reducing signaling through the nPKC and NF‐kB pathways. Importantly, our hierarchical, multidisciplinary approach led to a genetic signature of human longevity: the tightly connected and highly conserved PKC and NF‐κB signaling pathways.

Consistent with our data, decreased PKC and NF‐κB signaling promote longevity in model organisms (Curran & Ruvkun, 2007; Spindler et al., 2012; Zhang et al., 2013). The directional parallels between the effects of reduction in PKC and NF‐κB signaling on longevity of model organisms and those produced by the human longevity‐associated *PRKCH*, *NFKBIA*, and *CLU* regulatory variants are quite compelling. These results suggest that reductions in PKC and NF‐κB signaling are evolutionary‐conserved longevity mechanisms in humans and thus represent therapeutic targets for extending healthspan and lifespan.

## 2 RESULTS

### 2.1 Discovery of candidate longevity‐associated genes and variants by target capture sequencing

GWAS involving long‐lived individuals has predicted the presence of rare protective variants with strong effects in centenarians (Deelen et al., 2011, 2014; Nebel et al., 2011; Sebastiani et al., 2012). Indeed, a comprehensive candidate approach has demonstrated rare functional variants with protective effects are enriched in centenarians (Suh et al., 2008; Tazearslan et al., 2011), suggesting that centenarians may harbor individually rare, but collectively more common genetic variations in candidate longevity genes. Identifying such rare variants requires extensive sequencing analysis of a large centenarian cohort. To ensure identification of all possible longevity‐associated variants, including rare variants, we performed a hierarchical cost‐effective sequencing analysis of Ashkenazi Jewish centenarians and controls (Figure 1).

In stage 1, we selected 568 candidate genes (Table S1) implicated in cognitive function. These candidate genes included: (a) genes implicated in neurodegeneration such as AD and Parkinson's disease (PD); (b) AD‐related genes such as *APP* and *APOE* and interacting pathways; (c) genes implicated in cognitive function such as memory formation mechanism and neuronal receptors; and (d) genes implicated in lipid metabolism such as cholesterol synthesis, transport, and metabolism, e.g. *APOE* and *CETP*.

We then performed target capture sequencing (Capture‐seq) of the 568 candidate genes in 51 centenarians and 51 controls. Target regions of candidate genes included exons, exon‐intron junctions, and 2 kb proximal upstream regions to identify both coding and non‐coding variants. The Capture‐seq analysis identified a total of 13,574 variants in our candidate genes. The variants showed a similar distribution between centenarians and controls across all genomic regions (Table S2). The vast majority of the variants (74.9%, 10,161 out of 13,574) occurred in non‐coding regions with about 20% being previously unreported novel variants.

To identify candidate longevity‐associated genes enriched with rare variants, we performed a gene‐based association study that considered aggregates of rare variants in a gene region using SKAT analysis (Wu et al., 2011). We identified candidate longevity‐associated genes that surpass the threshold of nominal *p*‐value < 0.05 (Table S3). We further performed Ingenuity Pathway Analysis (IPA) to identify pathways that were enriched in significant genes (*p* < 0.05) from the SKAT analysis. We found that almost all of the top enriched pathways (*p* < 0.05) contained three protein kinase C (PKC) family genes (*PRKCB*, *PRKCH*, *PRKCI*) (Table S4).

### 2.2 Prioritization of candidate longevity‐associated genes by genotyping

We performed Fisher's exact test to identify variants associated with longevity. 457 variants were significantly associated with longevity (*p* <  0.05), among which the most significant variant was rs753381 in *PLCG1* gene (*p*‐value: 0.0003, Table S5). The majority of variants were rare, accounting for 58.8% (7,984 out of 13,547 variants) with minor allele frequency (MAF) less than 0.05. We then selected variants for genotyping in a larger number of individuals, i.e. 474 centenarians and 551 controls, to prioritize longevity‐associated genes, based on the following criteria: (a) association *p*‐values (*p* < 0.05); (b) rare variants (MAF < 0.05) enriched in either centenarians or controls; and (c) potentially functional variants including non‐synonymous, non‐sense and frameshift variants with MAF difference between the two groups.

We successfully genotyped a total of 222 variants using Sequenom MassARRAY iPLEX assays (Section 4). A total of 23 variants were significantly associated with longevity (nominal *p*‐value < 0.05), and 12 variants were enriched in centenarians as compared to controls (Tables S6 and S7). Notably, among the 12 variants, 8 variants were in genes involved in the PKC signaling pathway, including phospholipase C (PLC) family, Ca^2+^/calmodulin‐dependent protein kinase (CaMK), and EGF Receptor (*EGFR*). Together with the results from the SKAT analysis and pathway analysis (Tables S3 and S4), this result led us to focus on PKC family genes and genes involved in the pathways that interact with PKC such as PLC and CaMK signaling as top longevity‐associated genes among our initial 568 candidate genes implicated in cognitive function.

### 2.3 A second stage Capture‐seq analysis focused on PKC and PKC‐interacting pathway genes

In stage 2, we selected a comprehensive list of 217 genes (Table S8) acting in the PKC and PKC‐interacting pathways as candidate genes for the second Capture‐seq analysis. These included: (a) all known PKC and PLC family genes; (b) genes in PKC‐interacting pathways such as PKA, MAPK, and CaMK pathways; (c) genes in PKC‐upstream pathways such as neurotrophin receptors, neuronal receptors, EGF receptor, and inflammatory signaling; (d) PKC‐downstream genes such as those involved in NF‐κB, CREB, NFAT, and mTOR signaling; and (e) AD‐ and PD‐associated genes from GWAS. We also included significant genes (*p* < 0.05) from the stage 1 Capture‐seq study that were not in the PKC‐interacting pathways.

We performed the stage 2 second Capture‐seq analysis of the 217 genes in 450 centenarians and 500 controls. A total of 23,625 variants were discovered, among which 564 variants were significantly associated with longevity (*p* < 0.05) and the most significant variant was rs1092331 in *PRKCH* gene (*p*‐value: 0.0001, Table S9). The vast majority (89.57%, 21,160 out of 23,625) of these variants were in non‐coding regions and rare variants (81.05%, MAF <0.05, Table S10). Following SKAT analysis, we identified 22 genes that were associated with longevity (nominal *p*‐value < 0.05, Table 1).

**TABLE 1:** Longevity‐associated genes from the Stage 2 Capture‐seq in 450 centenarians and 500 controls (*p* < 0.05)

Gene	SKAT *p*‐value	Gene	SKAT *p*‐value
*NFKBIA*	0.0053	*NFATC3*	0.0294
*CLU*	0.0059	*PRKAR2A*	0.0297
*PRKCD*	0.0079	*PLCB1*	0.0319
*CREB1*	0.0097	*PDE4B*	0.0324
*PRKD3*	0.0137	*PPP2R1A*	0.0327
*EGFR*	0.0216	*MAPK4*	0.0334
*IL1B*	0.0216	*MAPK9*	0.0391
*PKN2*	0.0274	*ADCY1*	0.0424
*RELA*	0.0279	*IL6R*	0.0457
*NFKB1*	0.0287	*PRKCH*	0.0495
*NFATC2*	0.0293	*PLCG2*	0.0496

**FIGURE 2:**
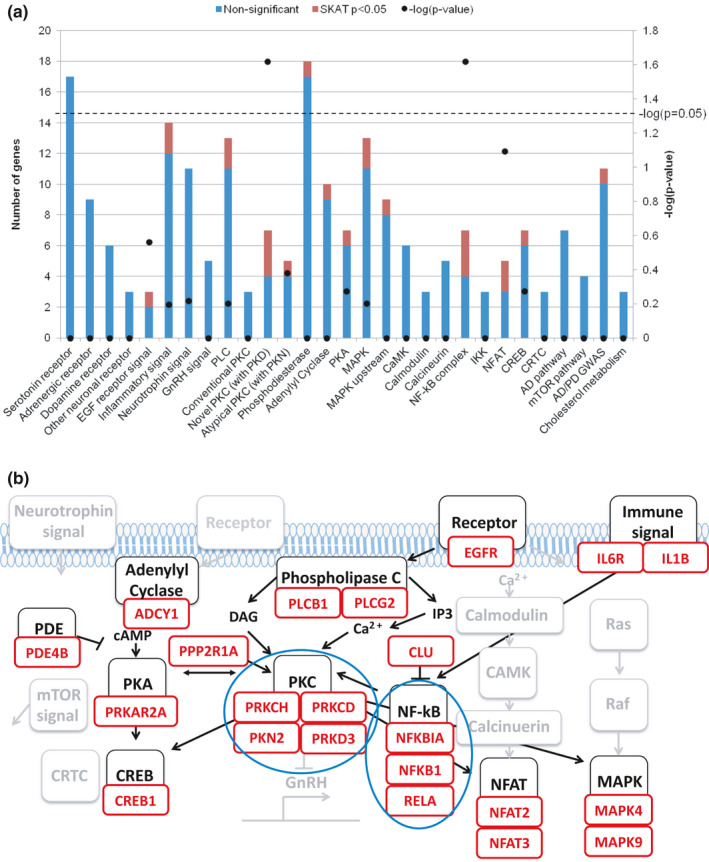
Sub‐pathways of PKC and PKC‐interacting pathways enriched with longevity‐associated genes from the Stage 2 Capture‐seq analyses. (a) 217 sub‐pathway genes included in the Stage 2 Capture‐seq were categorized and the number of longevity‐associated genes (SKAT *p* < 0.05) (red bar) and non‐significant genes (blue bar) in each sub‐pathway was shown. Enrichment analysis of longevity‐associated genes in each sub‐pathway was performed by Fisher's exact test and ‐log (*p*‐value) was indicated (black dot) on the graph. Dotted line indicated the threshold of significance of enrichment analysis (*p* = 0.05). (b) The pathway map indicates the PKC and PKC‐interacting genes and sub‐pathways. Longevity‐associated genes (red boxes, SKAT *p* < 0.05) were indicated below the sub‐pathways (black boxes). Blue circles indicate the top sub‐pathways enriched with longevity‐associated genes (Figure 2a). Gray boxes indicate sub‐pathways without longevity‐associated genes

To determine if any sub‐pathways of our PKC pathway‐centric candidate genes were enriched with longevity‐associated genes, we first sub‐categorized all candidate genes and then analyzed the enrichment ratio of longevity‐associated genes detected by SKAT analysis in each category (Figure 2a,b and Table S11). We found significant enrichment of longevity‐associated genes in two sub‐pathways (*p* < 0.05), the novel PKC family (*p* = 0.024) and the NF‐κB complex (*p* = 0.024) (Figure 2a,b).

### 2.4 In silico analysis to identify potentially functional variants in longevity‐associated genes

In Stage 3, we examined the functional relevance of the variants in the longevity‐associated genes in the PKC and PKC‐interacting pathways. For this purpose, we first conducted *in silico* analysis to prioritize potentially functional variants. For coding variants, we selected variants predicted to affect protein function and/or structure, including non‐synonymous variants, stop‐gain or stop‐loss variants, frameshift variants, and splicing variants. For non‐coding variants, we used RegulomeDB (http://regulomedb.org/) to identify potentially regulatory variants. For 3′ UTR variants, we took advantage of the starBase database (http://starbase.sysu.edu.cn/), which provides the experimental information of RNA‐binding protein such as Argonaute (Ago) protein from CLIP‐seq analysis. Thus, the genomic location of RNA‐binding protein in 3′ UTR is used to predict the microRNA‐binding sites based on experimental results (Section 4).

Using the predicted functional variants, we performed multiple SKAT analysis of all variants, coding variants, regulatory variants, or combined putative functional variants of the 22 longevity‐associated genes (Table 1). Along with SKAT analysis, which gives a weight to rare variants and considers both direction of enrichment in two groups, we also performed the SKAT‐O and SKAT‐C analyses that consider the directionality of rare variants enrichment in either one group and the effects of both common and rare variants, respectively.

**FIGURE 3:**
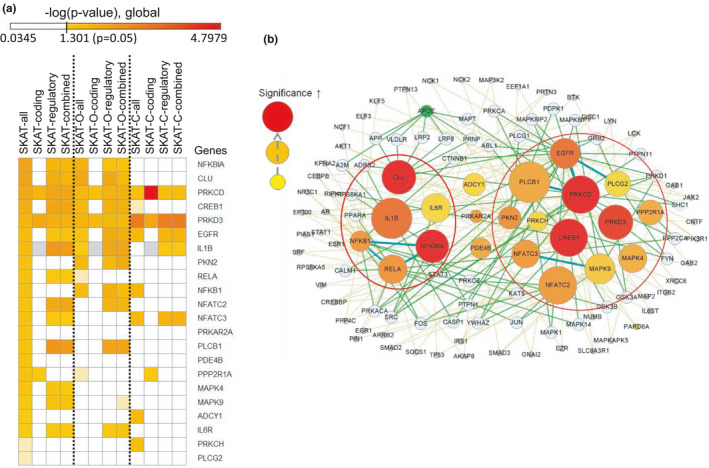
Gene‐based association studies with predicted functional variants and protein‐protein interaction networks for the 22 longevity‐associated genes. (a) The heatmap represents the significance of each gene‐based association analysis that includes SKAT, SKAT‐O, and SKAT‐C. Each analysis used all variants (‐all), predicted functional coding variants (‐coding), predicted functional regulatory variants (‐regulatory), or combined predicted functional coding and regulatory variants (‐combined). The genes were from the Stage 2 Capture‐seq analyses found to be longevity‐associated (SKAT *p* < 0.05, Table 1). Black dotted lines divide each SKAT, SKAT‐O, and SKAT‐C analysis. Gray indicates the absence of variants. (b) The network was generated with the 22 longevity‐associated genes (SKAT *p* < 0.05) from the Stage 2 Capture‐seq analyses. The color of circles indicates the significance from SKAT analysis and the size of circles indicates the significance of SKAT analysis with predicted functional variants. The direct interactions between proteins are indicated by the thick green lines. The red circles represent the top enriched pathways including NF‐κB and immune response (left) and PLC, PKC, EGF receptor, and downstream factors (right). Light blue medium‐sized circles indicate the proteins included in our candidate gene list without SKAT significance. Green circles indicate the ApoE protein, alleles of which have been associated with longevity and AD in multiple human genetic studies

Most of the 22 longevity‐associated genes detected by SKAT (SKAT‐all) also showed significant association with longevity when only predicted functional variants were analyzed by SKAT analysis (Figure 3a). Interestingly, most of the significance with functional variants was from the predicted functional effect of the regulatory variants. Notably, in SKAT‐C analysis—including both rare and common variants with only coding functional variants—the *PRKCD* gene showed the strongest *p*‐value: 0.000016. The information and the distribution of the *PRKCD* coding variants are indicated in Figure S1.

We generated protein‐protein interaction networks among the 22 longevity‐associated genes (Figure 3b). The generated networks highlighted PKC (*PRKCD*, *PRKD3*, *PKN2*, *PRKCH*) and their interaction with upstream PLC (*PLCB1*, *PLCG2*), EGF receptor (*EGFR*), and NF‐κB complex that are composed of the transcription factors p65 (*RELA*) and p50 (*NFKB1*) and the repressor IκBα (*NFKBIA*). In addition, the *CLU* gene that encodes clusterin/ApoJ protein showed overlapping interacting proteins with ApoE protein, alleles of which have been shown to associate with longevity and AD.

We also performed IPA ingenuity canonical pathway enrichment analysis to determine which functional pathways are enriched in longevity‐associated genes using a total of 217 candidate genes that we used for Stage 2 Capture‐seq analyses (Table S8). The most significantly enriched pathways are involved in immune response pathways such as pattern recognition receptor in recognition of bacteria and virus (*p* = 0.0005), the production of nitric oxide and reactive oxygen species in macrophages (*p* = 0.0027), and the role of macrophages, fibroblasts and endothelial cells in rheumatoid arthritis (*p* = 0.0039) (Table S12). The pathways also contain age‐related diseases such as signaling of atherosclerosis (*p* = 0.0045), type 2 diabetes (*p* = 0.0288), and Huntington's disease (*p* = 0.0467). Interestingly, we noticed that almost all of the top enriched pathways (*p* < 0.05) contained PKC family and NF‐κB complex genes (*PRKCD*, *PRKCH*, *PRKD3*, *RELA*, *NFKB1*, *NFKBIA*) (Table S12).

### 2.5 Functional analysis of regulatory variants in longevity‐associated genes in cell culture models

Recent studies have shown that the vast majority (>95%) of the variants detected by GWAS are non‐coding variants and 65% of non‐coding GWAS‐associated variants occur in enhancer regions (Hnisz et al., 2013), suggesting that gene regulatory changes contribute to inter‐individual differences in genetic risk. Similarly, most of the variants detected by the Stage 2 Capture‐seq analyses as well as those in the longevity‐associated genes (SKAT *p* < 0.05) were located in non‐coding regions of the genome (Table S10).

To ascertain biological significance of the longevity‐associated gene variants, we performed functional analysis of non‐coding variants with predicted regulatory potential using immune cell lines that may be functionally relevant based on the pathway analysis (Table S12). We selected non‐coding variants in the predicted regulatory regions of the two longevity‐associated genes *PRKCH* and *NFKBIA* (Table 2) in the top sub‐pathways (Figure 2a), the novel PKCs (*PRKCD*, *PRKCH*, *PRKD3*) and NF‐κB family members (*NFKBIA*, *NFKB1*, *RELA*). We also included non‐coding variants in the longevity‐associated *CLU* gene (Tables 1 and 2) because of its known function in regulation of PKC pathway through inhibition of the NF‐κB pathway (Santilli et al., 2003).

**TABLE 2:** Candidate regulatory variants in top prioritized longevity‐associated pathway genes for *in vitro* functional study

Gene	CHR	POS	ID	Region	MAF‐controls	MAF‐centenarians	*p*‐value	Regulome DB Score*	LD*
PRKCH	Chr14	61995673	rs2463117	Intronic	0.076	0.104	0.0496	**4**	DB/Seq
PRKCH	Chr14	61996018	rs17098729	Intronic	0.055	0.097	0.0015	7	DB/Seq
PRKCH	Chr14	61997226	rs1088680	exonic‐syn	0.078	0.122	0.0033	7	DB/Seq
PRKCH	Chr14	61997393	rs1088679	Intronic	0.065	0.109	0.0013	7	DB/Seq
PRKCH	Chr14	61997531	rs1092331	Intronic	0.063	0.112	0.0004	6	DB/Seq
CLU	Chr8	27467984	rs9331893	Intronic	0.008	0.028	0.0008	**4**	Seq.
CLU	Chr8	27464081	rs9331906	Intronic	0.008	0.023	0.0085	6	Seq.
NFKBIA	Chr14	35875417	**∙**	Upstream	0.001	0.012	0.0022	6	No
NFKBIA	Chr14	35874523	rs2233407	Upstream	0.023	0.050	0.0018	**4**	No
NFKBIA	Chr14	35874065	**∙**	Upstream	0.005	0.013	0.0847	**3a**	No

* Bold numbers indicate RegulomeDB scores less than or equal to 5 that are expected to be functional in our analysis. DB means database reported LD and Seq indicates the LD information from our sequencing result.

**FIGURE 4:**
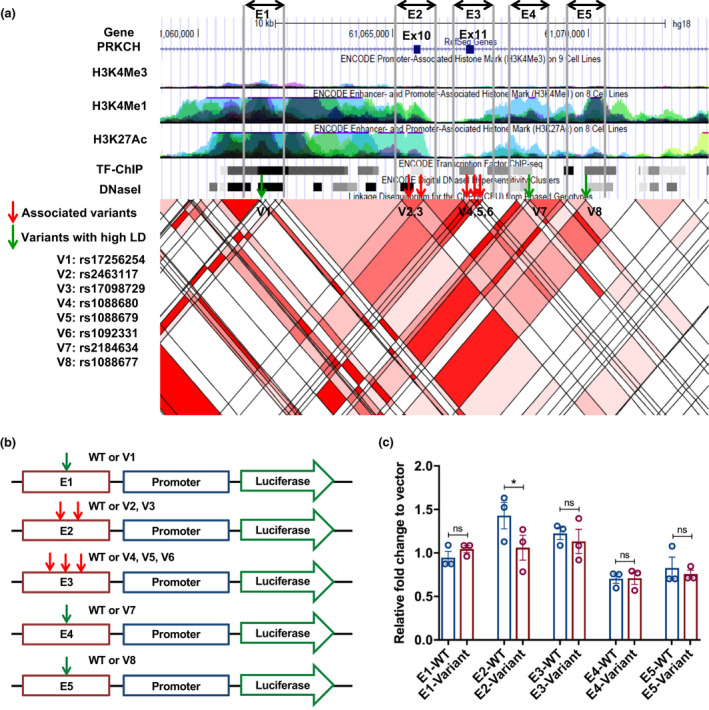
Functional analysis of the longevity‐associated regulatory variants in the *PRKCH* gene. (a) An overview of *PRKCH* gene region displayed in the UCSC genome browser. Red arrows indicate the longevity‐associated variants and green arrows indicate the variants in high LD. E1 to E5 regions indicate the putative enhancer regions harboring the variants to be investigated in our reporter assays. (b) A basic design of enhancer reporter constructs. (c) Enhancer reporter assays using E1 to E5 constructs along with a vector as a baseline control in U‐87 human glioblastoma cell line. The *y*‐axis indicates relative fold changes in reporter activity of the E1 to E5 constructs harboring either wild‐type (WT) or the longevity‐associated variants (*n* = 3). * indicates the *p*‐value less than 0.05 by *t*‐test

Due to extended linkage disequilibrium (LD) in the genome, multiple variants in an associated region can be identified as significant and hence identifying truly causal variants is highly challenging especially for non‐coding variants. For example, while an intronic variant, rs1092331, was the most significant variant (*p* = 0.0001) in the *PRKCH* gene by single variant association analysis (Table 2), it has multiple variants in LD (*R* ^2^ = 1, Figure 4a). We selected 5 candidate genomic regions (E1‐E5) harboring longevity‐associated variants (V2‐V6) or variants (V1, V7, and V8) in high LD that map to predicted enhancers based on well‐known histone marks (H3K4me1, H3K27ac) as well as a DNase I hypersensitive site (DHS) in the UCSC genome browser (Figure 4a).

To test the functional impact of the putative regulatory variants, we designed and performed enhancer reporter assays using the cloned fragments (E1‐E5) harboring wild‐type or variant sequences (Figure 4b) in the U‐87 MG human glioblastoma cell line. While E2 and E3 showed enhancer activities (Figure S2a), only the E2 region harboring V2 (rs2463117, *p* = 0.0496) and V3 (rs17098729, *p* = 0.0015) variants showed a modest but significant reduction in reporter activity as compared to E2 harboring the wild‐type sequence (Figure 4c). Thus, it was expected that centenarian‐enriched *PRKCH* gene variants may reduce PKC and NF‐κB signaling through decreased level of *PRCKH* gene expression.

**FIGURE 5:**
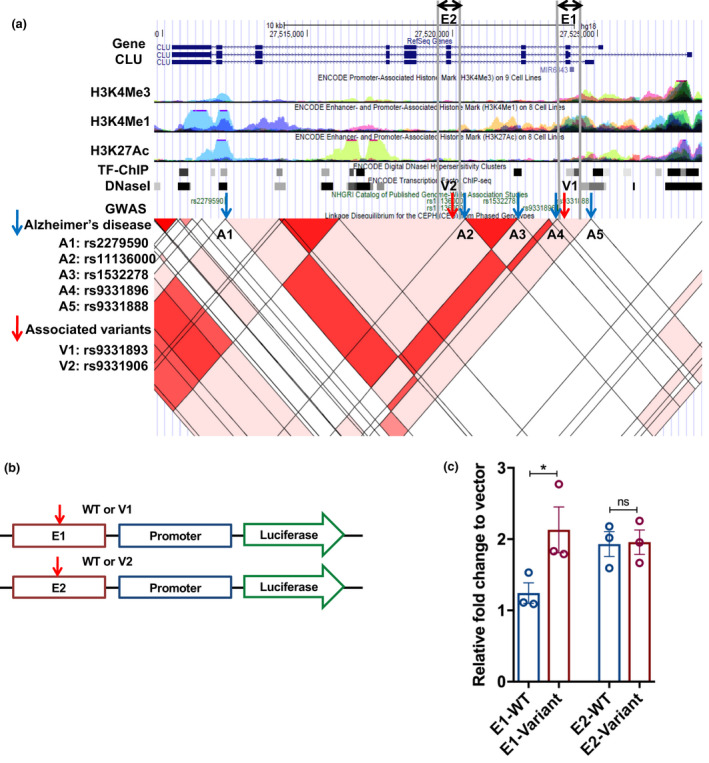
Functional analysis of the longevity‐associated regulatory variants in the *CLU* gene. (a) An overview of *CLU* gene region displayed in the UCSC genome browser. Red arrows indicate the longevity‐associated variants and blue arrowheads indicate the variants associated with risk of AD from GWAS. E1 and E2 indicate the putative enhancer regions harboring the variants to be investigated in reporter assays. (b) The design of the enhancer reporter constructs. (c) Enhancer reporter assays using E1, E2 constructs along with a vector as a baseline control in U‐87 human glioblastoma cell line. The *y*‐axis indicates relative fold changes in reporter activity of the E1 and E2 constructs harboring either wild‐type (WT) or the longevity‐associated variants (*n* = 3). * indicates the *p*‐value less than 0.05 by *t*‐test

For *CLU* gene (clusterin) variants (Figure S2b), we selected two candidate regulatory regions (E1 and E2) that map to predicted enhancers based on well‐known histone marks (H3K4me1, H3K27ac) as well as DHS in the UCSC genome browser. E1 and E2 harbor the longevity‐associated variants (V1: rs9331893 and V2: s9331906, respectively) (Figure 5a, and Table 2). We found that these variants are close to the previously reported variant (rs11136000) associated with decreased risk of AD (Harold et al., 2009; Lambert et al., 2009) (Figure 5a). Luciferase enhancer reporter assays using reporter constructs (Figure 5b) with and without the putative enhancer variants in U‐87 MG human glioblastoma cell line revealed that the E1 region with the variant rs9331893 had a significant increase in reporter activity compared to the wild‐type sequence (Figure 5c). Given the inhibitory function of clusterin on the NF‐κB pathway through the stabilization of IκBs (Santilli et al., 2003), the increased level of *CLU* expression caused by the longevity‐associated variant may diminish PKC and NF‐κB signaling.

**FIGURE 6:**
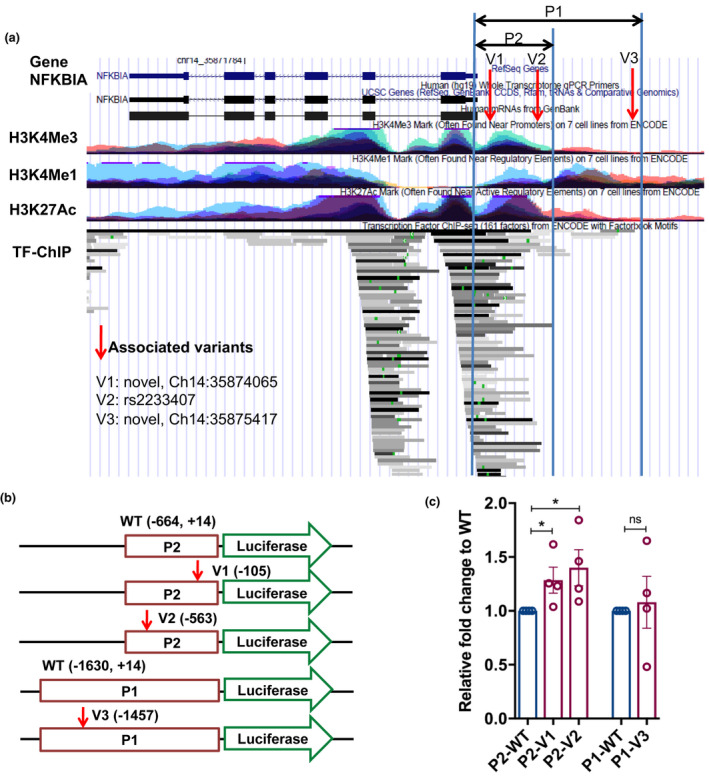
Functional analysis of the longevity‐associated regulatory variants in the *NFKBIA* gene. (a) An overview of *NFKBIA* gene region displayed in the UCSC genome browser. Red arrows indicate the longevity‐associated variants. P1 and P2 indicate the putative promoter regions harboring the variants to be investigated in reporter assays. (b) A basic design of promoter reporter constructs. (c) Promoter reporter assays using P1, P2 constructs in THP‐1 human monocytic cell line. The *y*‐axis indicates relative fold changes in reporter activity of the constructs harboring either wild‐type (WT) or variants (V1, V2, V3) in P1, P2 regions (*n* = 4). * indicates the *p*‐value less than 0.05 by Mann‐Whitney *U* test

The *NFKBIA* gene encoding the NF‐κB repressor IκBα was the most significantly associated gene by SKAT analysis (*p* = 0.0053, Table 1). We found three centenarian‐enriched rare variants in the proximal promoter region within 2 kb upstream of transcription start site (Figure 6a). The variants were two novel variants, chr14:35875417 (TAGAG>T) and chr14:35874065 (G>A), and rs2233407 (Table 2). We found little evidence of LD amongst these variants. To investigate possible changes in the promoter activity conferred by these variants, we generated two promoter reporter constructs (Figure 6b): P1 contained the 1.6 kb proximal promoter region that harbors a centenarian‐enriched variant, V3 (−1457, chr14:35875417 (TAGAG>T) and P2 contained the 0.6 kb proximal promoter region harboring two centenarian‐enriched variants, V1 (−105, chr14:35874065 (G>A) and V2 (−563, rs2233407). The P2 region overlaps with a predicted promoter based on the well‐known histone mark, H3K4me3, as well as numerous transcription factor bindings (Figure 6a). We performed the *in vitro* reporter assays in THP‐1 human monocytic cell line using the P1 and P2 constructs with and without the longevity‐associated variants. We found that both chr14:35874065 (G>A) (V1) and rs2233407 (V2) variants in the P1 region caused a modest, but significant increase in promoter activities as compared to wild‐type (Figure 6c). Since higher expression of IκBα results in a decrease in NF‐κB transcriptional activity, taken together, these results suggest that the longevity‐associated variants reduce the signaling of nPKC and NF‐κB pathways by modulating expression of their cognate genes.

## 3 DISCUSSION

The ultimate goal of this study was to establish if genes implicated in maintaining cognitive function into old age also potentially impact human longevity. From the outset, this study focused on identification of rare, functional variants based on the results from previous GWAS that clearly suggesting the presence of rare, protective variants enriched in long‐lived individuals (Deelen et al., 2011, 2014; Nebel et al., 2011; Sebastiani et al., 2012). Our main hypothesis was that rare functional variants in genes implicated in cognitive function may contribute to human longevity. To test this hypothesis, we conducted a 3‐stage systematic study (Figure 1). In stage 1 and 2 Capture‐seq analysis, we identified 22 longevity‐associated genes (Table 1), which were found to be enriched in the nPKC family and the NF‐κB complex by sub‐pathway analysis (Figure 2a,b and Table S11). By integrating *in silico* analysis and *in vitro* reporter assays in the Stage 3, we identified functional regulatory variants in three longevity‐associated genes, *PRKCH*, *NFKBIA*, and *CLU* that modulated the expression of the cognate genes. The directionality of the effects on gene expression conferred by the longevity‐associated variants suggested that these variants decrease signaling through the nPKC and NF‐κB pathways, thereby contributing to the longevity phenotype.

Our study represents by far the largest study for a comprehensive genetic analysis of candidate genes, involving 474 centenarians and 551 controls, in search of longevity‐associated genes and variants. It should be noted that defining and selecting control individuals pose a unique challenge in any case‐control study of human longevity (Sebastiani et al., 2017). This issue becomes more challenging in the identification of rare variants associated with longevity. We have been tackling this critical issue by taking a strategic study design. First, we have been studying Ashkenazi Jewish centenarians and controls, a genetically isolated population which is more powerful in identifying rare causal variants not only for Mendelian disease genes but also complex traits such as Type 2 Diabetes (T2D) (Steinthorsdottir et al., 2014). Second, we have performed functional analyses to confirm the genetic and biological significance of top variants.

In the Stage 1 discovery genetic study, single variant association and gene‐based association studies with a moderate sample size could detect some genetic signatures at the pathway level. However, it was critical to validate the association in a large number of individuals because only a small portion of significant variants and genes were validated. 6.35% (4 out of 63) of significant variants and 8.7% (2 out of 23) of genes in the Stage 1 Capture‐seq remained significant in genotyping and Stage 2 Capture‐seq analysis, respectively (Table S7 and Table 1). The Stage 2 Capture‐seq analysis identified many non‐coding region variants, which were mainly intronic (50.96%) and in the upstream region (13.36%), as well as exonic region variants (10.38%). Compared to all variants, a higher proportion of longevity‐associated variants were identified in intronic (53.90%) and upstream (16.13%) regions, while lower in the exonic region (9.04%). (Table S10). This suggests that variants in non‐coding regions such as upstream and intronic regions may play an important role in longevity by regulation of their cognate gene expression as was implicated in GWAS studies (Hnisz et al., 2013).

In addition, rare variants (MAF < 0.05) were mainly located in non‐coding region including intronic (49.87%), upstream (13.20%), and 3′ UTR (17.34%) region (Table S10). Therefore, we performed gene‐based association analysis, SKAT, including the non‐coding region, considering their predicted regulatory role of gene expression. With longevity‐associated 22 genes (Table 1) identified by SKAT analysis including all discovered variants, we also performed multiple SKAT analyses to consider a unique genetic signature of each gene in regards to distribution of variants in genomic region (coding or non‐coding), frequency (rare or common), and directionality (one directional or bi‐directional) (Figure 3a). By this analysis, the overview of unique characteristics of variants in each longevity‐associated gene could be predicted. This highlights the importance of setting criteria about which variants need to be included in gene‐based association study based on their predicted function.

We defined nominal *p*‐values of <0.05 as significant throughout the study in order to include all possible candidate longevity‐associated genes and variants to avoid possible type 2 errors. This was particularly important for Stage 1 study with a limited number of centenarians (*n* = 51) and controls (*n* = 51). In stage 2 with a larger number of individuals (500 centenarians and 450 controls), we validated the candidate genes from the stage 1 and could identify the top candidate functional variants. To complement the statistical limitation and boost our confidence, we performed the functional analysis of the *PRKCH*, *NFKB1A*, and *CLU* gene variants. These genes belong to the top candidate pathway, nPKC and NF‐kB pathway (Figure 1), which is known to play evolutionary conserved roles in modulating lifespan in model organisms (Curran & Ruvkun, 2007; Spindler et al., 2012; G. Zhang et al., 2013). Indeed, our data indicate that the top candidate variants exert functional effects in regulation of the cognate target gene expression in a direction that is mechanistically parallel to what has been known to promote longevity in the model organisms. Our results suggest that in a well‐designed genetic association study of human longevity such as ours, one may not be overly concerned about the inflated type I error rate due to multiple testing as long as (a) the prioritization is not based on *p*‐values only, but also on other genetic and biological information; and (b) the top variants are confirmed in functional follow‐up studies by directly testing for the impact of longevity‐associated variants.

Association studies generally leave open the question of whether an associated genetic variant is functionally important or can serve only as a genetic marker with the functional locus co‐inherited with the associated variant. To minimize possible errors and spurious associations, we took an approach to assess the functional relevance of gene variations by directly testing their impact in *in vitro* functional analyses (Tazearslan et al., 2012). For functional studies, we chose physiologically relevant human cell lines for the longevity‐associated genes implicated in immune function (Table S12), the U‐87 MG human glioblastoma cell line and THP‐1 human monocytic cell line, because different cell types have different regulatory mechanisms of gene expression. We chose three longevity‐associated genes and their centenarian‐enriched variants in the PKC and NF‐κB pathways, *PRKCH*, *NFKBIA*, and *CLU*. All but one of the candidate longevity‐associated variants in the *PRKCH*, *NFKBIA*, and *CLU* genes were located in non‐coding regions of the genome (Table 2). RegulomeDB analysis identified that 4 out of 10 variants were equal or less than 5, which meet the criteria used for the SKAT analysis with functional regulatory variants. In studies using cell model, all of the 4 predicted functional variants affected luciferase reporter activity while the other variants did not change the activity (Figures 4c, 5c, and 6c), suggesting that functional regulatory variants were predicted well. The longevity‐associated *CLU* variants are close to variants associated with the decreased risk of AD detected by multiple GWAS (Harold et al., 2009; Lambert et al., 2009) (Figure 5a). An AD‐associated variant, rs11136000, in the *CLU* gene was shown to be correlated with reduced incidence of AD as well as increased expression of the CLU1 isoform in the human brain (Ling et al., 2012). Thus, we hypothesize that the longevity‐associated intronic variant contributes to longevity and healthy cognitive aging by increasing *CLU* gene expression.

In this study, we identified a genetic signature of decreased signaling of PKC and NF‐κB pathways that may contribute to human longevity. Interestingly, animal model studies showed that decreased signaling of PKC and NF‐kB pathway increased lifespan. Decrease of the PKC pathway activity (Spindler et al., 2012), novel PKC pathway including PKCη (*PRKCH*) (Monje et al., 2011), PKCδ (*PRKCD*) (Curran & Ruvkun, 2007), and PKD3 (*PRKD3*) (Feng et al., 2007) have been implicated in longevity in model organisms. Negatively affecting expression of PLC family genes, which are directly upstream of PKC, is also reported to increase lifespan, especially PLCβ (Kawli et al., 2010) and PLCγ with EGF receptor (Iwasa et al., 2010). In agreement with the animal studies, *PLCB1*, *PLCG2*, and *EGFR* were found to be associated with longevity in our study. NF‐κB complex genes act downstream of PKC. In our study, we identified the 3 major components of NF‐κB complex, *NFKBIA* (IκBα), *NFKB1* (p50), and *RELA* (p65), among the top 22 longevity‐associated genes. In model organisms, inhibition of NF‐κB activity delayed aging and increased lifespan, while enhanced activity accelerated aging (Kawahara et al., 2009; Tilstra et al., 2012; Zhang et al., 2013). Deficiency of *CLU* gene product in aged mice increases the severity of immune response mediated myocarditis in heart (McLaughlin et al., 2000) and glomerulopathy (Rosenberg et al., 2002), two age‐related diseases. Indeed, in *Drosophila*, over‐expression of human clusterin (*CLU*) increased stress resistance and extended lifespan (Lee et al., 2012). This suggests that this conserved pathway may influence longevity in humans, analogous as to what has been demonstrated in model organisms.

It is possible that the genes and signals we identified are population‐specific and may need validations in different human populations (Franceschi et al., 2020). However, our data suggest that functional variants with the same directional impact on the evolutionary conserved genes as the variants found in our study may play a similar role in different human populations, providing evidence for therapeutic modulation.

With the identification of rare variants enriched in long‐lived individuals, there is now a pressing need to functionally validate the variants to understand the mechanisms by which these variants contribute to longevity in humans. An integrated analysis using experimental and computational approaches in parallel will help elucidate the molecular mechanisms of functional variants, which then be further tested in cell and animal models (Zhang et al., 2020). The longevity‐associated variants in the context of evolutionary conservation provide unique opportunities to translate genetic discoveries to therapeutic modulation.

In summary, by taking a hierarchical, comprehensive candidate approach, we found a genetic signature of human longevity in the hyperconnected PKC and NF‐κB pathways among genes implicated in cognitive function. Our results suggest that reduction in PKC and NF‐κB signaling may promote longevity in humans as has been observed in model organisms. Further studies will ultimately reveal the novel role of PKC and NF‐κB signaling in longevity and maintenance of cognitive function in human populations and provide important mechanistic insights into the molecular basis of aging.

## 4 EXPERIMENTAL PROCEDURES

### 4.1 Study subjects and sample collection

Our study group consisted of 474 Ashkenazi Jewish (AJ) centenarians and 551 AJ controls that were collected as part of a Longevity Genes Project by Dr. Nir Barzilai of the Albert Einstein College of Medicine. A centenarian was defined as a healthy individual living independently at 95 years of age or older and a control was defined as an individual without a family history of unusual longevity. The control group consisted largely of spouses of the offspring of centenarians. All blood samples were rapidly processed to obtain DNA at the General Clinical Research Center of the Albert Einstein College of Medicine. Informed written consent was obtained in accordance with the policy of the Committee on Clinical Investigations of the Albert Einstein College of Medicine.

For the initial discovery by Capture‐seq of our candidate genes, we used genomic DNA extracted from immortalized B‐lymphocytes; 51 centenarians of 95–109 age range (mean: 99.9 ± 3.8) and 51 controls of 48–81 age range (mean: 66.37 ± 8.79) were used. For Capture‐seq and genotyping experiments, whole genome amplified DNA was used for which the template DNA was obtained directly from blood samples and amplified using illustra GenomiPhi V2 DNA Amplification kits (GE healthcare Life Sciences). We studied 450 centenarians of 95–110 age range (mean: 98.21 ± 2.9) and 550 controls of 43–93 age range (mean: 73.14 ± 9.1) for the Capture‐seq and 474 centenarians of 95–110 age range (mean 98.33 ± 2.92), and 551 controls of 39–94 age range (mean 74.44 ± 8.77) for the genotyping experiments.

### 4.2 Target selection and generation of customized target capture for the Stage 1 Capture‐seq

Genes implicated in cognitive function and related pathway genes were broadly selected as candidate genes for our study. For the initial discovery study by Capture‐seq, 568 genes were selected based on a comprehensive literature and database search. For AD and PD genes, we used database AlzGene (http://www.alzgene.org), PDgene (www.pdgene.org), and GWAS catalog (http://www.genome.gov/gwastudies). For the following Stage 2 Capture‐seq analyses of prioritized PKC‐interacting pathway genes, we selected genes from a comprehensive literature search. The complete list of initial 568 candidate genes for the Stage 1 Capture‐seq is provided in Table S1.

We designed a customized Agilent SureSelect in‐solution target capture to enrich our candidate genes. For each selected gene locus, we included a 2‐kb upstream region of the transcription start site, all exons, and 20 bp of each exon‐intron junction. We used the Agilent eArray (http://earray.chem.agilent.com/earray/) to design and assess coverage across the target genomic regions of bait libraries. We designed an assay of 568 candidate genes for the target capture sequencing experiment to maximize the sequencing capacity (Han et al., 2013). For the 568 genes, design efficiency from eArray was 99%.

### 4.3 Generation of customized target capture for the Stage 2 Capture‐seq

For the first Stage 2 Capture‐seq, we designed a customized Nimblegen SeqCap EZ Choice library (Roche) for target capture to enrich 30 candidate genes (Table S8). We included a 2‐kb upstream region of the transcription start site, all exons, and exon‐intron junction of target genes. We used the NimbleDesign (https://design.nimblegen.com/nimbledesign) with ‘Max close match’ set to 5 to design and assess coverage across the target genomic regions of bait libraries. For the 30 genes, design efficiency from eArray was 97.9%.

For the second Stage 2 Capture‐seq, we designed target capture for 187 candidate genes (Table S8) with NimbleDesign as in the first Stage 2 Capture‐seq, except for using ‘Preferred close match’ set to 3 and ‘Maximum close match’ set to 17 for update and change of system. Design efficiency from eArray was 96.3%.

### 4.4 Genotyping

The iPLEX MassArray assays (Sequenom) were designed in Genomics Shared Facility at Albert Einstein College of Medicine. A total of 333 SNPs were designed for iPLEX assays in multiplexing groups. 222 SNPs were successfully assayed based on the high quality of peaks visualized in the MassARRAY^®^ Typer software. Using the iPLEX assays, genotyping was performed in 474 centenarians and 551 controls in the Genomics Shared Facility at Albert Einstein College of Medicine. The association analyses were performed using SNP & Variation suite version 7.6.11 (Golden helix) and JMP Genomics (SAS). Statistical differences among groups were assessed by the Fisher's exact test, for both allelotype and genotype distribution comparisons. A two‐tailed *p*‐value of < 0.05 was considered significant. Due to the low frequency of the rare variants, we could not consider sex as a covariate in our statistical analysis.

### 4.5 Rare variation association analysis

We used a R package called “SKAT” for SKAT, SKAT‐O, and SKAT‐C analysis as methods for rare variant association from both the Stage 1 and 2 Capture‐seq (Wu et al., 2011). The SKAT analysis for the Stage 2 Capture‐seq was performed by using minor allele frequency (MAF) of variants in each pool instead of allele counts in each individual using modified R script. The script was generated from personal communication with a person who developed SKAT analysis (Dr. Seunggeun Lee). This method is an alternative way to perform SKAT in Pool‐seq result with expense of underestimation of *p*‐value since individual genotype information was lost in a pool.

### 4.6 Selection of predicted functional regulatory variants

As the criteria for selecting predicted functional regulatory variants, we selected variants that have the RegulomeDB score equal to or less than 5, which overlapped with any least one transcription factor binding or DHS. In addition, if at least one experiment reported the Ago protein binding in an overlapping region of variants, we selected the variants predicted to be functional in 3′ UTR region.

### 4.7 Cell culture condition

Human glioblastoma cell line U‐87 MG, and human monocyte cell line THP‐1 cells were purchased from American Type Culture Collection (ATCC). U‐87 MG cells were maintained in Dulbecco's modified Eagle medium (DMEM), THP‐1 cells were maintained in RPMI. Both of them were supplemented with 10% fetal bovine serum (FBS) and 1% penicillin‐streptomycin at 37ºC in a humidified 5% CO_2_.

### 4.8 Generation of luciferase reporter constructs and assay

Genomic DNAs from individuals who harbored *PRKCH*, *CLU* intronic heterozygote variants, and reference sequences were used to amplify the intronic regions by PCR. PCR was performed using Phusion high fidelity DNA polymerase (New England BioLabs, Inc., Massachusetts). The amplified products were cloned into pGL3 vector with SV40 promoter (Promega), and the sequence of the construct was verified. For *NFKBIA* upstream variants, genomic DNA of an individual harboring reference sequence were used for PCR amplification and cloned into promoter‐less pGL4 vector (Promega). Mutagenesis was performed to generate constructs with desired variants using QuikChange II XL Site‐Directed Mutagenesis Kit (Agilent).

For functional studies of the variants in *PRKCH* or *CLU* genes, 1 × 10^5^ of U‐87 MG cells were cultured on 24‐well plates 24 hours prior to transfection, and transfected with 300 ng consisting of luciferase reporter plasmids and pRL‐TK (Promega) in a ratio of 5:1. For functional studies of *NFKBIA* gene variants, 2 × 10^4^ of THP‐1 cell were cultured on 96‐well plates 24 hours prior to transfection, and transfected with 110 ng consisting of luciferase reporter plasmids and pRL‐CMV (Promega) in a ratio of 10:1. Transfections were performed using X‐tremeGENE HP transfection reagents (Roche) according to the manufacturer's instructions. 24 hours after transfection, cells were harvested and the luciferase activities were measured using a Dual‐Luciferase Reporter Assay System (Promega) on a microplate reader (BioTek). Enhancer and promoter activities were normalized with Renilla luciferase activities.

### 4.9 Protein‐protein interaction network generation

We used the Human Protein Reference Database (HPRD, release 9), and High‐quality INTeractomes (HINT) as the background network (with all self‐interactions ignored) and our selected top genes as the “seeds”. To generate the sub‐network using Cytoscape program, we searched for all shortest paths between every pair of the “seeds”. The resultant shortest paths together constitute the sub‐network. To make the sub‐network simple and informative, we ignored any shortest paths longer than the smallest shortest path length with which all the selected top genes would be included in the sub‐network.

## CONFLICT OF INTEREST

The authors declare that they have no competing interests.

## AUTHOR CONTRIBUTIONS

NJS and YS conceived and designed the experiments. SR and JH performed the experiments. SR, JH, TNK, QZ, SL, ZZ analyzed the data. GA and NB collected and provided AJ samples. SR, LJN, PDR, and YS contributed to the writing of the manuscript. All authors read and approved the final manuscript.

## Supporting information

Supplementary Material

Table S5

Table S7

Table S9

## Data Availability

Sequencing data that support the findings of this study have been deposited in NCBI Sequence Read Archive (PRJNA669033, PRJNA669034, and PRJNA669037).

## References

[acel13362-bib-0001] Adams, E. R. , Nolan, V. G. , Andersen, S. L. , Perls, T. T. , & Terry, D. F. (2008). Centenarian offspring: Start healthier and stay healthier. Journal of the American Geriatrics Society, 56(11), 2089–2092. 10.1111/j.1532-5415.2008.01949.x 18811609 PMC2892731

[acel13362-bib-0002] Ailshire, J. A. , Beltrán‐Sánchez, H. , & Crimmins, E. M. (2015). Becoming centenarians: Disease and functioning trajectories of older US Adults as they survive to 100. Journals of Gerontology. Series A, Biological Sciences and Medical Sciences, 70(2), 193–201. 10.1093/gerona/glu124 PMC4311187 25136001

[acel13362-bib-0003] Andersen, S. L. , Sebastiani, P. , Dworkis, D. A. , Feldman, L. , & Perls, T. T. (2012). Health span approximates life span among many supercentenarians: Compression of morbidity at the approximate limit of life span. Journals of Gerontology. Series A, Biological Sciences and Medical Sciences, 67(4), 395–405. 10.1093/gerona/glr223 PMC3309876 22219514

[acel13362-bib-0004] Barzilai, N. , Atzmon, G. , Derby, C. A. , Bauman, J. M. , & Lipton, R. B. (2006). A genotype of exceptional longevity is associated with preservation of cognitive function. Neurology, 67(12), 2170–2175. 10.1212/01.wnl.0000249116.50854.65 17190939

[acel13362-bib-0005] Barzilai, N. , Gabriely, I. , Gabriely, M. , Iankowitz, N. , & Sorkin, J. D. (2001). Offspring of centenarians have a favorable lipid profile. Journal of the American Geriatrics Society, 49(1), 76–79. 11207846 10.1046/j.1532-5415.2001.49013.x

[acel13362-bib-0006] Christensen, K. , Johnson, T. E. , & Vaupel, J. W. (2006). The quest for genetic determinants of human longevity: Challenges and insights. Nature Reviews Genetics, 7(6), 436–448. 10.1038/nrg1871 PMC2726954 16708071

[acel13362-bib-0007] Curran, S. P. , & Ruvkun, G. (2007). Lifespan regulation by evolutionarily conserved genes essential for viability. PLoS Genetics, 3(4), e56. 10.1371/journal.pgen.0030056 17411345 PMC1847696

[acel13362-bib-0008] Deelen, J. , Beekman, M. , Uh, H.‐W. , Broer, L. , Ayers, K. L. , Tan, Q. , Kamatani, Y. , Bennet, A. M. , Tamm, R. , Trompet, S. , Guðbjartsson, D. F. , Flachsbart, F. , Rose, G. , Viktorin, A. , Fischer, K. , Nygaard, M. , Cordell, H. J. , Crocco, P. , van den Akker, E. B. , … Slagboom, P. E. (2014). Genome‐wide association meta‐analysis of human longevity identifies a novel locus conferring survival beyond 90 years of age. Human Molecular Genetics, 23(16), 4420–4432. 10.1093/hmg/ddu139 24688116 PMC4103672

[acel13362-bib-0009] Deelen, J. , Beekman, M. , Uh, H.‐W. , Helmer, Q. , Kuningas, M. , Christiansen, L. , Kremer, D. , van der Breggen, R. , Suchiman, H. E. D. , Lakenberg, N. , van den Akker, E. B. , Passtoors, W. M. , Tiemeier, H. , van Heemst, D. , de Craen, A. J. , Rivadeneira, F. , de Geus, E. J. , Perola, M. , van der Ouderaa, F. J. , … Slagboom, P. E. (2011). Genome‐wide association study identifies a single major locus contributing to survival into old age; the APOE locus revisited. Aging Cell, 10(4), 686–698. 10.1111/j.1474-9726.2011.00705.x 21418511 PMC3193372

[acel13362-bib-0010] Dubal, D. B. , Yokoyama, J. S. , Zhu, L. , Broestl, L. , Worden, K. , Wang, D. , Sturm, V. E. , Kim, D. , Klein, E. , Yu, G.‐Q. , Ho, K. , Eilertson, K. E. , Yu, L. , Kuro‐o, M. , De Jager, P. L. , Coppola, G. , Small, G. W. , Bennett, D. A. , Kramer, J. H. , … Mucke, L. (2014). Life extension factor klotho enhances cognition. Cell Reports, 7(4), 1065–1076. 10.1016/j.celrep.2014.03.076 24813892 PMC4176932

[acel13362-bib-0011] Feng, H. , Ren, M. , Chen, L. , & Rubin, C. S. (2007). Properties, regulation, and in vivo functions of a novel protein kinase D: Caenorhabditis elegans DKF‐2 links diacylglycerol second messenger to the regulation of stress responses and life span. Journal of Biological Chemistry, 282(43), 31273–31288. 10.1074/jbc.M701532200 17728253

[acel13362-bib-0012] Franceschi, C. , Garagnani, P. , Olivieri, F. , Salvioli, S. , & Giuliani, C. (2020). The contextualized genetics of human longevity: JACC focus seminar. Journal of the American College of Cardiology, 75(8), 968–979. 10.1016/j.jacc.2019.12.032 32130932

[acel13362-bib-0013] Han, J. , Ryu, S. , Moskowitz, D. M. , Rothenberg, D. , Leahy, D. J. , Atzmon, G. , Barzilai, N. , & Suh, Y. (2013). Discovery of novel non‐synonymous SNP variants in 988 candidate genes from 6 centenarians by target capture and next‐generation sequencing. Mechanisms of Ageing and Development, 134, 478–485. 10.1016/j.mad.2013.01.005 23376243 PMC3787996

[acel13362-bib-0014] Harold, D. , Abraham, R. , Hollingworth, P. , Sims, R. , Gerrish, A. , Hamshere, M. L. , Pahwa, J. S. , Moskvina, V. , Dowzell, K. , Williams, A. , Jones, N. , Thomas, C. , Stretton, A. , Morgan, A. R. , Lovestone, S. , Powell, J. , Proitsi, P. , Lupton, M. K. , Brayne, C. , … Williams, J. (2009). Genome‐wide association study identifies variants at CLU and PICALM associated with Alzheimer's disease. Nature Genetics, 41(10), 1088–1093. 10.1038/ng.440 19734902 PMC2845877

[acel13362-bib-0015] Herskind, A. M. , McGue, M. , Holm, N. V. , Sorensen, T. I. , Harvald, B. , & Vaupel, J. W. (1996). The heritability of human longevity: A population‐based study of 2872 Danish twin pairs born 1870–1900. Human Genetics, 97(3), 319–323. 8786073 10.1007/BF02185763

[acel13362-bib-0016] Hitt, R. , Young‐Xu, Y. , Silver, M. , & Perls, T. (1999). Centenarians: The older you get, the healthier you have been. Lancet, 354(9179), 652. 10.1016/S0140-6736(99)01987-X. 10466675

[acel13362-bib-0017] Hnisz, D. , Abraham, B. J. , Lee, T. I. , Lau, A. , Saint‐André, V. , Sigova, A. A. , Hoke, H. A. , & Young, R. A. (2013). Super‐enhancers in the control of cell identity and disease. Cell, 155(4), 934–947. 10.1016/j.cell.2013.09.053. 24119843 PMC3841062

[acel13362-bib-0018] Iwasa, H. , Yu, S. , Xue, J. , & Driscoll, M. (2010). Novel EGF pathway regulators modulate *C. elegans* healthspan and lifespan via EGF receptor, PLC‐gamma, and IP3R activation. Aging Cell, 9(4), 490–505. 10.1111/j.1474-9726.2010.00575.x 20497132 PMC5859306

[acel13362-bib-0019] Jonsson, T. , Atwal, J. K. , Steinberg, S. , Snaedal, J. , Jonsson, P. V. , Bjornsson, S. , Stefansson, H. , Sulem, P. , Gudbjartsson, D. , Maloney, J. , Hoyte, K. , Gustafson, A. , Liu, Y. , Lu, Y. , Bhangale, T. , Graham, R. R. , Huttenlocher, J. , Bjornsdottir, G. , Andreassen, O. A. , … Stefansson, K. (2012). A mutation in APP protects against Alzheimer's disease and age‐related cognitive decline. Nature, 488(7409), 96–99. 10.1038/nature11283 22801501

[acel13362-bib-0020] Kawahara, T. L. , Michishita, E. , Adler, A. S. , Damian, M. , Berber, E. , Lin, M. , & Chua, K. F. (2009). SIRT6 links histone H3 lysine 9 deacetylation to NF‐kappaB‐dependent gene expression and organismal life span. Cell, 136(1), 62–74. 10.1016/j.cell.2008.10.052 19135889 PMC2757125

[acel13362-bib-0021] Kawli, T. , Wu, C. , & Tan, M. W. (2010). Systemic and cell intrinsic roles of Gqalpha signaling in the regulation of innate immunity, oxidative stress, and longevity in *Caenorhabditis elegans* . Proceedings of the National Academy of Sciences of the United States of America, 107(31), 13788–13793. 10.1073/pnas.0914715107 20647387 PMC2922217

[acel13362-bib-0022] Kliegel, M. , Moor, C. , & Rott, C. (2004). Cognitive status and development in the oldest old: A longitudinal analysis from the Heidelberg Centenarian Study. Archives of Gerontology and Geriatrics, 39(2), 143–156. 10.1016/j.archger.2004.02.004 15249151

[acel13362-bib-0023] Lambert, J.‐C. , Heath, S. , Even, G. , Campion, D. , Sleegers, K. , Hiltunen, M. , Combarros, O. , Zelenika, D. , Bullido, M. J. , Tavernier, B. , Letenneur, L. , Bettens, K. , Berr, C. , Pasquier, F. , Fiévet, N. , Barberger‐Gateau, P. , Engelborghs, S. , De Deyn, P. , Mateo, I. , … Amouyel, P. (2009). Genome‐wide association study identifies variants at CLU and CR1 associated with Alzheimer's disease. Nature Genetics, 41(10), 1094–1099. 10.1038/ng.439 19734903

[acel13362-bib-0024] Lee, Y. N. , Shim, Y. J. , Kang, B. H. , Park, J. J. , & Min, B. H. (2012). Over‐expression of human clusterin increases stress resistance and extends lifespan in Drosophila melanogaster. Biochemical and Biophysical Research Communications, 420(4), 851–856. 10.1016/j.bbrc.2012.03.087 22465014

[acel13362-bib-0025] Ling, I. F. , Bhongsatiern, J. , Simpson, J. F. , Fardo, D. W. , & Estus, S. (2012). Genetics of clusterin isoform expression and Alzheimer's disease risk. PLoS One, 7(4), e33923. 10.1371/journal.pone.0033923 22506010 PMC3323613

[acel13362-bib-0026] McGue, M. , Vaupel, J. W. , Holm, N. , & Harvald, B. (1993). Longevity is moderately heritable in a sample of Danish twins born 1870–1880. The Journal of Gerontology, 48(6), B237–244. 8227991 10.1093/geronj/48.6.b237

[acel13362-bib-0027] McLaughlin, L. , Zhu, G. , Mistry, M. , Ley‐Ebert, C. , Stuart, W. D. , Florio, C. J. , Groen, P. A. , Witt, S. A. , Kimball, T. R. , Witte, D. P. , Harmony, J. A. K. , & Aronow, B. J. (2000). Apolipoprotein J/clusterin limits the severity of murine autoimmune myocarditis. Journal of Clinical Investigation, 106(9), 1105–1113. 10.1172/JCI9037 PMC301413 11067863

[acel13362-bib-0028] Monje, J. M. , Brokate‐Llanos, A. M. , Perez‐Jimenez, M. M. , Fidalgo, M. A. , & Munoz, M. J. (2011). pkc‐1 regulates daf‐2 insulin/IGF signalling‐dependent control of dauer formation in *Caenorhabditis elegans* . Aging Cell, 10(6), 1021–1031. 10.1111/j.1474-9726.2011.00747.x 21933341

[acel13362-bib-0029] Murabito, J. M. , Yuan, R. , & Lunetta, K. L. (2012). The search for longevity and healthy aging genes: Insights from epidemiological studies and samples of long‐lived individuals. Journals of Gerontology. Series A, Biological Sciences and Medical Sciences, 67(5), 470–479. 10.1093/gerona/gls089 PMC3326242 22499766

[acel13362-bib-0030] Nebel, A. , Kleindorp, R. , Caliebe, A. , Nothnagel, M. , Blanché, H. , Junge, O. , Wittig, M. , Ellinghaus, D. , Flachsbart, F. , Wichmann, H.‐E. , Meitinger, T. , Nikolaus, S. , Franke, A. , Krawczak, M. , Lathrop, M. , & Schreiber, S. (2011). A genome‐wide association study confirms APOE as the major gene influencing survival in long‐lived individuals. Mechanisms of Ageing and Development, 132(6–7), 324–330. 10.1016/j.mad.2011.06.008 21740922

[acel13362-bib-0031] Perls, T. (2004). Dementia‐free centenarians. Experimental Gerontology, 39(11–12), 1587–1593. 10.1016/j.exger.2004.08.015 15582273

[acel13362-bib-0032] Rosenberg, M. E. , Girton, R. , Finkel, D. , Chmielewski, D. , Barrie, A. , Witte, D. P. , Zhu, G. , Bissler, J. J. , Harmony, J. A. K. , & Aronow, B. J. (2002). Apolipoprotein J/clusterin prevents a progressive glomerulopathy of aging. Molecular and Cellular Biology, 22(6), 1893–1902. 11865066 10.1128/MCB.22.6.1893-1902.2002 PMC135592

[acel13362-bib-0046] Ruby J. G. , Wright K. M. , Rand K. A. , Kermany A. , Noto K. , Curtis D. , Varner N. , Garrigan D. , Slinkov D. , Dorfman I. , Granka J. M. , Byrnes J. , Myres N. , Ball C. (2018). Estimates of the Heritability of Human Longevity Are Substantially Inflated due to Assortative Mating. Genetics, 210(3), 1109–1124. 10.1534/genetics.118.301613 30401766 PMC6218226

[acel13362-bib-0033] Sanders, A. E. , Wang, C. , Katz, M. , Derby, C. A. , Barzilai, N. , Ozelius, L. , & Lipton, R. B. (2010). Association of a functional polymorphism in the cholesteryl ester transfer protein (CETP) gene with memory decline and incidence of dementia. JAMA, 303(2), 150–158. 10.1001/jama.2009.1988 20068209 PMC3047443

[acel13362-bib-0034] Santilli, G. , Aronow, B. J. , & Sala, A. (2003). Essential requirement of apolipoprotein J (clusterin) signaling for IkappaB expression and regulation of NF‐kappaB activity. Journal of Biological Chemistry, 278(40), 38214–38219. 10.1074/jbc.C300252200 12882985

[acel13362-bib-0035] Sebastiani, P. , Bae, H. , Gurinovich, A. , Soerensen, M. , Puca, A. , & Perls, T. T. (2017). Limitations and risks of meta‐analyses of longevity studies. Mechanisms of Ageing and Development, 165(Pt B), 139–146. 10.1016/j.mad.2017.01.008 28143747 PMC5533653

[acel13362-bib-0036] Sebastiani, P. , Solovieff, N. , DeWan, A. T. , Walsh, K. M. , Puca, A. , Hartley, S. W. , Melista, E. , Andersen, S. , Dworkis, D. A. , Wilk, J. B. , Myers, R. H. , Steinberg, M. H. , Montano, M. , Baldwin, C. T. , Hoh, J. , & Perls, T. T. (2012). Genetic signatures of exceptional longevity in humans. PLoS One, 7(1), e29848. 10.1371/journal.pone.0029848 22279548 PMC3261167

[acel13362-bib-0037] Spindler, S. R. , Li, R. , Dhahbi, J. M. , Yamakawa, A. , & Sauer, F. (2012). Novel protein kinase signaling systems regulating lifespan identified by small molecule library screening using Drosophila. PLoS One, 7(2), e29782. 10.1371/journal.pone.0029782 22363408 PMC3282711

[acel13362-bib-0038] Steinthorsdottir, V. , Thorleifsson, G. , Sulem, P. , Helgason, H. , Grarup, N. , Sigurdsson, A. , Helgadottir, H. T. , Johannsdottir, H. , Magnusson, O. T. , Gudjonsson, S. A. , Justesen, J. M. , Harder, M. N. , Jørgensen, M. E. , Christensen, C. , Brandslund, I. , Sandbæk, A. , Lauritzen, T. , Vestergaard, H. , Linneberg, A. , … Stefansson, K. (2014). Identification of low‐frequency and rare sequence variants associated with elevated or reduced risk of type 2 diabetes. Nature Genetics, 46(3), 294–298. 10.1038/ng.2882 24464100

[acel13362-bib-0039] Suh, Y. , Atzmon, G. , Cho, M.‐O. , Hwang, D. , Liu, B. , Leahy, D. J. , Barzilai, N. , & Cohen, P. (2008). Functionally significant insulin‐like growth factor I receptor mutations in centenarians. Proceedings of the National Academy of Sciences of the United States of America, 105(9), 3438–3442. 10.1073/pnas.0705467105 18316725 PMC2265137

[acel13362-bib-0040] Tazearslan, C. , Cho, M. , & Suh, Y. (2012). Discovery of functional gene variants associated with human longevity: Opportunities and challenges. Journals of Gerontology. Series A, Biological Sciences and Medical Sciences, 67(4), 376–383. 10.1093/gerona/glr200 PMC3309874 22156437

[acel13362-bib-0041] Tazearslan, C. , Huang, J. , Barzilai, N. , & Suh, Y. (2011). Impaired IGF1R signaling in cells expressing longevity‐associated human IGF1R alleles. Aging Cell, 10(3), 551–554. 10.1111/j.1474-9726.2011.00697.x 21388493 PMC3094477

[acel13362-bib-0042] Tilstra, J. S. , Robinson, A. R. , Wang, J. , Gregg, S. Q. , Clauson, C. L. , Reay, D. P. , & Robbins, P. D. (2012). NF‐kappaB inhibition delays DNA damage‐induced senescence and aging in mice. The Journal of Clinical Investigation, 122(7), 2601–2612. 10.1172/JCI45785 22706308 PMC3386805

[acel13362-bib-0043] Wu, M. C. , Lee, S. , Cai, T. , Li, Y. , Boehnke, M. , & Lin, X. (2011). Rare‐variant association testing for sequencing data with the sequence kernel association test. American Journal of Human Genetics, 89(1), 82–93. 10.1016/j.ajhg.2011.05.029 21737059 PMC3135811

[acel13362-bib-0044] Zhang, G. , Li, J. , Purkayastha, S. , Tang, Y. , Zhang, H. , Yin, Y. , & Cai, D. (2013). Hypothalamic programming of systemic ageing involving IKK‐beta, NF‐kappaB and GnRH. Nature, 497(7448), 211–216. 10.1038/nature12143 23636330 PMC3756938

[acel13362-bib-0045] Zhang, Z. D. , Milman, S. , Lin, J.‐R. , Wierbowski, S. , Yu, H. , Barzilai, N. , Gorbunova, V. , Ladiges, W. C. , Niedernhofer, L. J. , Suh, Y. , Robbins, P. D. , & Vijg, J. (2020). Genetics of extreme human longevity to guide drug discovery for healthy ageing. Nature Metabolism, 2(8), 663–672. 10.1038/s42255-020-0247-0 PMC7912776 32719537

