# Supplementary material for: Genetic signature of human longevity in PKC and NF‐κB signaling
Source: Aging Cell. 2021 Jul 1;20(7):e13362. doi: 10.1111/acel.13362 (PMC8282271; doi:10.1111/acel.13362)
Supplement: Supplementary file 1 — Supplementary Material [file ACEL-20-e13362-s003.docx]

**
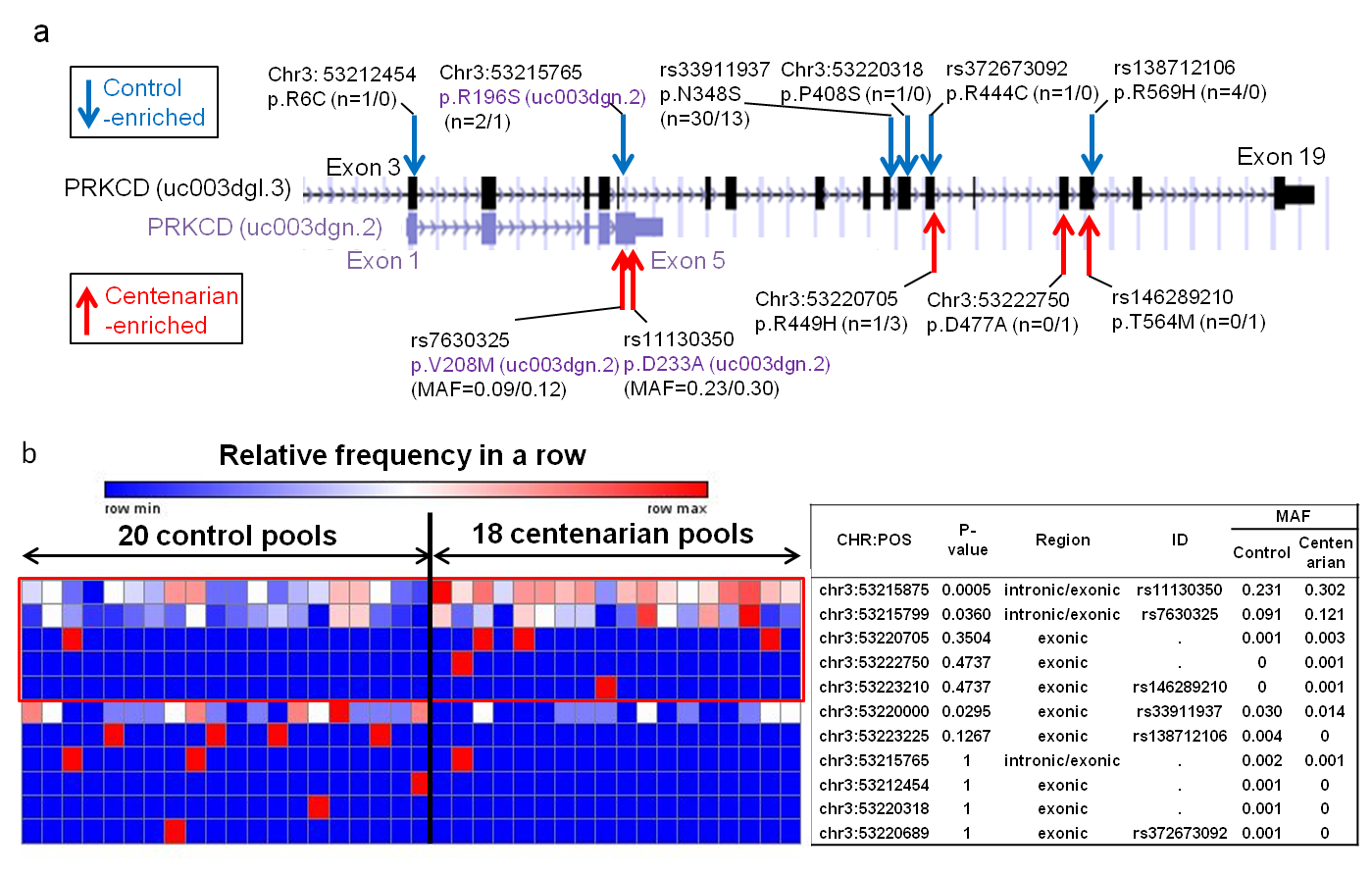
**

(b)

(a)

**Supplementary Figure 1. Location and distribution of coding variants in longevity-associated *PRKCD* gene**

(a) The diagram indicates the gene structure of *PRKCD* gene from UCSC genome browser and location and nature of discovered coding variants. PRKCD (uc0003dgl.3) and PRKCD (uc0003dgn.2) indicate gene ID of UCSC gene. (b) The heatmap represents distribution of *PRKCD* coding variants in centenarians (n=450) and controls (n=500). The red box indicates variants enriched in centenarians, while the lower part below the red box indicates control-enriched variants.


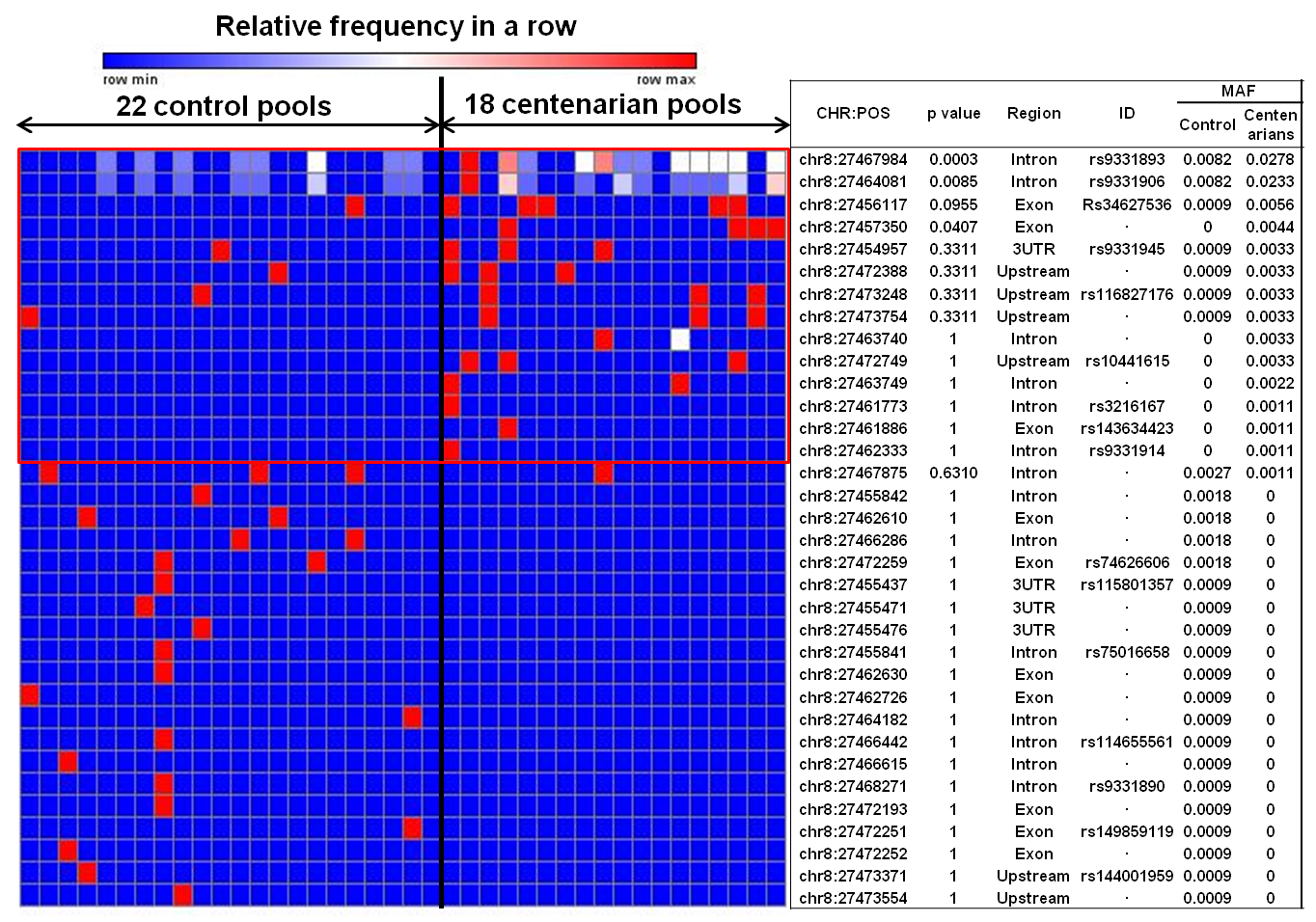

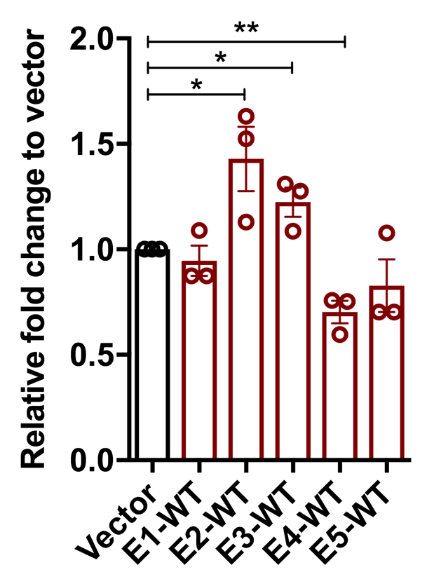
**Supplementary Figure 2. Enhancer activity of the regions used for functional study in the PRKCH gene and distribution of rare CLU gene variants**

(b)

(a)

(a) Enhancer reporter assays using wild-type E1 to E5 constructs along with an empty vector as a baseline control in U87 human glioblastoma cell line. The y-axis indicates relative fold changes in reporter activity of the E1 to E5 constructs harboring WT compared to vector (n=3). * indicates the p-value less than 0.05 and ** indicates the p-value less than 0.01 by t-test. (b) The heatmap represents the pattern of rare CLU gene variants discovered by Capture-seq analysis of 450 centenarians and 550 controls. Each column represents each pool with 25 individuals and each row represents each variant. The table describes information of each variant located in a matched row. p-values are based on Fisher’s exact test.

**Supplementary Table 1. The list of 568 candidate genes implicated in impacting cognitive function for the Stage 1 Capture-seq**

| A2M | BMP1 | CHRNA5 | F12 | GRIK1 | IL1B | MAPK8IP2 | PCED1B | PRKCZ | TFCP2L1 |
| --- | --- | --- | --- | --- | --- | --- | --- | --- | --- |
| A2ML1 | BMPR1A | CHRNA6 | F13A1 | GRIK2 | IL33 | MAPK8IP3 | PCK1 | PRNP | TFPI |
| ABCA1 | BMPR1B | CHRNA7 | FARP1 | GRIK3 | IL8 | MAPK9 | PCYT1A | PRUNE2 | TFPI2 |
| ABCB1 | BMPR2 | CHRNA9 | FASN | GRIK4 | INSIG1 | MAPT | PDE1B | PSEN1 | THBS1 |
| ABCG1 | BST1 | CHRNB1 | FBXO7 | GRIK5 | INSIG2 | MBTPS1 | PDE1C | PSEN2 | THBS2 |
| ABCG4 | C3 | CHRNB2 | FDFT1 | GRIN1 | ITGA3 | MBTPS2 | PDXK | PSENEN | THRA |
| ABCG8 | C5AR1 | CHRNB3 | FGF20 | GRIN2A | ITGA5 | MCPH1 | PEX5 | PTCH1 | TNK1 |
| ABL1 | CALB1 | CHRNB4 | FITM2 | GRIN2B | ITGB1BP1 | MMAB | PGBD1 | PTCH2 | TPI1 |
| ACADM | CALHM1 | CHRND | FOS | GRIN2C | ITM2A | MME | PICALM | PTCHD1 | TRIM8 |
| ACAT1 | CALM1 | CHRNE | FRK | GRIN2D | ITM2B | MMP13 | PIK3C2A | PTCHD2 | TRPC4AP |
| ACE | CALM2 | CHRNG | FRZB | GRINA | ITM2C | MMP9 | PIK3C2B | PTCHD3 | TSHZ1 |
| ADAM10 | CALM3 | CLEC3B | FYN | GRM1 | KAT5 | MSR1 | PIK3C2G | PTGS1 | TSHZ2 |
| ADAM17 | CALML3 | CLSTN1 | FZD1 | GRM2 | KIAA0101 | MTHFR | PIK3C3 | PTGS2 | TSHZ3 |
| ADAM19 | CAMK1D | CPT2 | FZD2 | GRM3 | KLC1 | MTTP | PIKFYVE | PTH | UCHL1 |
| ADAM9 | CAMK1G | CR1 | FZD8 | GRM4 | LCAT | MVK | PINK1 | PZP | USP24 |
| ADIPOQ | CAMK2A | CR1L | GABBR2 | GRM5 | LDLR | MYD88 | PIP5KL1 | RAP1A | VLDLR |
| ADORA1 | CAMK2B | CR2 | GABRA1 | GRM6 | LDLRAD1 | NAA15 | PITX3 | RAPGEF1 | VNN1 |
| ADORA2A | CAMK2D | CREB1 | GABRA2 | GRM7 | LDLRAD2 | NAA16 | PLA2G2A | RARA | WNT1 |
| ADORA2B | CAMK2G | CREB3 | GABRA3 | GRM8 | LDLRAD3 | NAE1 | PLA2G4A | RARB | WNT3A |
| ADORA3 | CAMK2N1 | CREB5 | GABRA4 | GRN | LDLRAP1 | NARG2 | PLA2G6 | RASD1 | WNT4 |
| ADRA1A | CAMK2N2 | CRKL | GABRA5 | GSK3A | LEF1 | NCSTN | PLAT | RBP1 | YWHAB |
| ADRA1B | CAMK4 | CST3 | GABRA6 | GSTM1 | LEP | NDE1 | PLAU | RELN | YWHAE |
| ADRA1D | CAMKK1 | CTNNB1 | GABRB1 | HDAC1 | LIPA | NDEL1 | PLAUR | RXRG | YWHAG |
| ADRA2A | CAMKK2 | CUBN | GABRB2 | HDAC10 | LIPC | NEDD4L | PLCB1 | SAA1 | YWHAH |
| ADRA2B | CAMSAP1 | CYP24A1 | GABRD | HDAC11 | LIPG | NEDD9 | PLCB2 | SAA2 | YWHAQ |
| ADRA2C | CAND1 | CYP2D6 | GABRE | HDAC2 | LNX1 | NGFR | PLCB3 | SAA4 | YWHAZ |
| ADRB1 | CASP1 | CYP46A1 | GABRG1 | HDAC3 | LPL | NOS1AP | PLCB4 | SCARB1 | ZFP2 |
| ADRB2 | CASP10 | DAB1 | GABRG2 | HDAC4 | LRAT | NPC1 | PLCD1 | SERPINA1 | ZNF189 |
| ADRB3 | CASP12 | DAB2 | GABRG3 | HDAC5 | LRP1 | NPC1L1 | PLCD3 | SERPINC1 |  |
| AFTPH | CASP14 | DAPK1 | GABRP | HDAC6 | LRP10 | NPC2 | PLCD4 | SERPIND1 |  |
| ALB | CASP2 | DCTN1 | GABRQ | HDAC8 | LRP11 | NPY | PLCE1 | SERPINE1 |  |
| ANKRA2 | CASP3 | DDX1 | GABRR1 | HMG20A | LRP12 | NPY1R | PLCG1 | SERPING1 |  |
| AP2A1 | CASP4 | DECR1 | GABRR2 | HMGCR | LRP1B | NR1D1 | PLCG2 | SET |  |
| APBA2 | CASP5 | DGAT1 | GABRR3 | HP | LRP2 | NR1H2 | PLCH1 | SFN |  |
| APBB1 | CASP6 | DKK1 | GALNT2 | HRH1 | LRP2BP | NR1H3 | PLCH2 | SHC1 |  |
| APBB1IP | CASP7 | DLG2 | GALP | HRH2 | LRP3 | NR1H4 | PLCL1 | SHC2 |  |
| APBB2 | CASP8 | DLG4 | GAPDHS | HRH3 | LRP4 | NTRK1 | PLTP | SHC3 |  |
| APBB3 | CASP9 | DNAH1 | GBA | HRH4 | LRP5 | NTRK2 | PM20D1 | SHC4 |  |
| APC | CAV1 | DRD1 | GC | HSP90B1 | LRP5L | NTRK3 | PON1 | SHCBP1 |  |
| APC2 | CAV2 | DRD2 | GHRL | HTR1A | LRP6 | NUCKS1 | PPARA | SHH |  |
| APH1A | CAV3 | DRD3 | GHSR | HTR1B | LRP8 | OPRD1 | PPARD | SLC2A2 |  |
| APH1B | CCR2 | DRD4 | GIGYF2 | HTR1D | LRPAP1 | OPRK1 | PPARG | SLC41A1 |  |
| APLP1 | CD33 | DRD5 | GIPC1 | HTR1E | LRRFIP2 | OPRL1 | PPM1E | SLC6A3 |  |
| APLP2 | CDHR1 | DTYMK | GLI1 | HTR1F | LRRK2 | OPRM1 | PPM1F | SMO |  |
| APOC1 | CDHR2 | DVL1 | GLI2 | HTR2A | LTF | OTC | PPP2CA | SNCA |  |
| APOC2 | CDK5 | DVL2 | GLI3 | HTR2B | MAGI2 | P2RX2 | PPP2CB | SNCB |  |
| APOC3 | CDK5R1 | DVL3 | GLI4 | HTR2C | MAOB | PAFAH1B1 | PPP2R1A | SNCG |  |
| APOE | CDK5R2 | ECE1 | GLIS1 | HTR3A | MAPK1 | PARK7 | PPP2R1B | SORCS1 |  |
| APOH | CETP | EFNA5 | GLRA1 | HTR3B | MAPK10 | PCDH1 | PPP2R2A | SORCS2 |  |
| APPBP2 | CFL1 | EGFR | GLRA2 | HTR3C | MAPK11 | PCDH10 | PPP2R2B | SORCS3 |  |
| ARMCX3 | CHRM1 | ENAH | GLRA3 | HTR3D | MAPK12 | PCDH11X | PPP2R2C | SORL1 |  |
| ARSB | CHRM2 | ENTPD7 | GLRA4 | HTR3E | MAPK13 | PCDH12 | PPP2R2D | SORT1 |  |
| ATF2 | CHRM3 | EPHB1 | GLRB | HTR4 | MAPK14 | PCDH15 | PRKCA | SPATA1 |  |
| ATF4 | CHRM4 | EPHB2 | GM2A | HTR5A | MAPK15 | PCDH17 | PRKCB | SRC |  |
| ATP13A2 | CHRM5 | EPHB3 | GOLM1 | HTR6 | MAPK3 | PCDH18 | PRKCD | SREBF2 |  |
| ATXN1 | CHRNA1 | EPHB4 | GPR3 | HTR7 | MAPK4 | PCDH19 | PRKCE | SYNJ2BP |  |
| AXIN1 | CHRNA10 | EPHB6 | GRIA1 | HTRA2 | MAPK6 | PCDH20 | PRKCG | TCF7 |  |
| AXIN2 | CHRNA2 | ERBB2 | GRIA2 | HTT | MAPK7 | PCDH7 | PRKCH | TF |  |
| BACE2 | CHRNA3 | EVL | GRIA3 | IDE | MAPK8 | PCDH8 | PRKCI | TFAM |  |
| BDNF | CHRNA4 | F11 | GRIA4 | IL1A | MAPK8IP1 | PCDH9 | PRKCQ | TFCP2 |  |

**Supplementary Table 2. Characteristics and numbers of variants in 568 candidate genes by the Stage 1 Capture-seq in 51 centenarians and 51 controls**

| Genomic  Region | SNPs in databases | | Novel SNPs | | Total SNPs | | |
| --- | --- | --- | --- | --- | --- | --- | --- |
|  | Controls | Cente-  narians | Controls | Cente-  narians | Controls | Cente-  narians | All |
| downstream | 114 | 108 | 16 | 22 | 130 | 130 | 153 |
| exonic | 2334 | 2311 | 360 | 341 | 2694 | 2652 | 3357 |
| exonic;splicing | 30 | 32 | 11 | 8 | 41 | 40 | 56 |
| intergenic | 716 | 693 | 158 | 155 | 874 | 848 | 1026 |
| intronic | 2157 | 2126 | 487 | 452 | 2644 | 2578 | 3155 |
| ncRNA | 113 | 109 | 31 | 32 | 144 | 141 | 167 |
| splicing | 12 | 14 | 2 | 4 | 14 | 18 | 20 |
| Upstream (2kb) | 1031 | 1039 | 289 | 252 | 1320 | 1291 | 1529 |
| UTR3 | 2180 | 2178 | 642 | 599 | 2822 | 2777 | 3416 |
| UTR5 | 438 | 437 | 135 | 152 | 573 | 589 | 695 |
| Total | 9125 | 9047 | 2131 | 2017 | 11256 | 11064 | 13574 |

* dbSNP 135 has been used for database

**Supplementary Table 3. Candidate longevity-associated genes from SKAT analysis (p < 0.05) from the Stage 1 Capture-seq, ranked by p value.**

| Gene | SKAT p-value | Gene | SKAT p-value |
| --- | --- | --- | --- |
| *YWHAZ* | 0.0008 | *CHRND* | 0.0327 |
| ***PRKCB*** | 0.0033 | *OPRK1* | 0.0328 |
| *LDLRAD1* | 0.0044 | *DRD1* | 0.0331 |
| *NUCKS1* | 0.0114 | *LIPG* | 0.0332 |
| ***PRKCH*** | 0.0120 | *SLC41A1* | 0.0358 |
| *HTR3B* | 0.0178 | *LRPAP1* | 0.0362 |
| *CLSTN1* | 0.0192 | *PDE1B* | 0.0366 |
| *GRIK5* | 0.0207 | *PLCD4* | 0.0376 |
| *NOS1AP* | 0.0207 | *CASP5* | 0.0381 |
| *CHRM5* | 0.0211 | *PPP2R1A* | 0.0397 |
| *TFCP2* | 0.0223 | *FRZB* | 0.0407 |
| *PTGS1* | 0.0240 | *ITGB1BP1* | 0.0449 |
| *NTRK1* | 0.0244 | *CAMK2B* | 0.0460 |
| *FARP1* | 0.0268 | ***PRKCI*** | 0.0460 |
| *CREB5* | 0.0297 | *CHRNG* | 0.0471 |
| *PSEN2* | 0.0320 |  |  |

* Bold indicates genes in PKC family genes

**Supplementary Table 4. Top Ingenuity canonical pathways enriched with candidate longevity-associated genes detected by SKAT analysis (p < 0.05) in the Stage 1 Capture-seq**

| **Ingenuity Canonical Pathways** | **p-value** | **Genes in pathway** |
| --- | --- | --- |
| Protein kinase A signaling | 0.005 | ***PRKCB, PRKCH, PRKCI****, PDE1B, YWHAZ, CREB5,*  *PLCD4, CAMK2B* |
| Mechanisms of viral exit from host cells | 0.009 | ***PRKCB, PRKCH, PRKCI*** |
| Dopamine-DARPP32 feedback in camp signaling | 0.010 | ***PRKCB, PRKCH, PRKCI****, PPP2R1A, DRD1,*  *CREB5, PLCD4* |
| Melatonin signaling | 0.015 | ***PRKCB, PRKCH, PRKCI****, PLCD4, CAMK2B* |
| mTOR signaling | 0.025 | ***PRKCB, PRKCH, PRKCI****, PPP2R1A* |
| nNOS signaling in neurons | 0.025 | ***PRKCB, PRKCH, PRKCI****, NOS1AP* |
| P2Y purigenic receptor signaling pathway | 0.025 | ***PRKCB, PRKCH, PRKCI****, CREB5, PLCD4* |
| P70s6K signaling | 0.026 | ***PRKCB, PRKCH, PRKCI****, PPP2R1A, YWHAZ, PLCD4* |
| GNRH signaling | 0.032 | ***PRKCB, PRKCH, PRKCI****, CREB5, CAMK2B* |
| Breast cancer regulation by stathmin1 | 0.032 | ***PRKCB, PRKCH, PRKCI****, PPP2R1A, CAMK2B* |
| Calcium-induced T lymphocyte apoptosis | 0.034 | ***PRKCB, PRKCH, PRKCI*** |
| CREB signaling in neurons | 0.034 | ***PRKCB, PRKCH, PRKCI****, GRIK5, CREB5, PLCD4,*  *CAMK2B* |
| ErbB4 signaling | 0.038 | ***PRKCB, PRKCH, PRKCI****, PSEN2* |
| GαA signaling | 0.038 | *DRD1, OPRK1, CREB5, CHRM5* |
| VDR/RXR activation | 0.041 | ***PRKCB, PRKCH, PRKCI*** |
| Androgen signaling | 0.041 | ***PRKCB, PRKCH, PRKCI*** |
| Gap junction signaling | 0.044 | ***PRKCB, PRKCH, PRKCI****, DRD1, PLCD4* |
| Fcγ receptor-mediated phagocytosis in macrophages and monocytes | 0.048 | ***PRKCB, PRKCH, PRKCI*** |

* Bold indicates genes in PKC family genes

**Supplementary Table 5. The longevity-associated variants detected by Fisher’s exact test (p < 0.05) in the Stage 1 Capture-seq**

- Separate file

**Supplementary Table 6. The longevity-associated significant variants (p < 0.05) by genotyping of selected variants from the Stage 1 Capture-seq in 474 centenarians and 551 controls**

| Gene | CHR | POS | Minor Allele | Major Allele | ID | Region | Exonic | Capture-seq (51 vs 51) | | | Genotyping (551 vs 474) | | | 1000  Gen. |
| --- | --- | --- | --- | --- | --- | --- | --- | --- | --- | --- | --- | --- | --- | --- |
|  |  |  |  |  |  |  |  | MAF-controls | MAF-  cent. | P-value | MAF-controls | MAF-  cent. | P-value | MAF |
| **PRKCH** | chr14 | 61997226 | T | C | rs1088680 | exonic | Syn | 0.039 | 0.137 | 0.0239 | 0.076 | 0.119 | 0.0011 | 0.303 |
| **PRKCH** | chr14 | 61786976 | T | C | rs12878845 | upstream |  | 0.363 | 0.284 | 0.2948 | 0.350 | 0.286 | 0.0022 | 0.491 |
| **PLCG2** | chr16 | 81942028 | C | G | rs72824905 | exonic | NS | 0.020 | 0.000 | 0.4993 | 0.013 | 0.002 | 0.0094 | 0.005 |
| HTR3B | chr11 | 113802600 | C | T | rs45597437 | intronic | - | 0.069 | 0.022 | 0.1771 | 0.038 | 0.018 | 0.0107 | 0.048 |
| NOS1AP | chr1 | 162313597 | C | T | rs347278 | intronic | - | 0.049 | 0.000 | 0.0594 | 0.041 | 0.021 | 0.0114 | 0.067 |
| **PRKCI** | chr3 | 169985829 | AT | A | . | intronic | - | 0.010 | 0.033 | 0.3424 | 0.012 | 0.028 | 0.0141 | - |
| ADRA1A | chr8 | 26614201 | C | T | . | UTR3 | - | 0.039 | 0.000 | 0.1240 | 0.009 | 0.001 | 0.0145 | - |
| CAMK2B | chr7 | 44256712 | G | A | rs148569268 | down  stream | - | 0.010 | 0.078 | 0.0268 | 0.026 | 0.047 | 0.0147 | 0.007 |
| **PLCG2** | chr16 | 81979943 | A | C | rs140070662 | intronic | - | 0.000 | 0.029 | 0.2463 | 0.001 | 0.009 | 0.0148 | 0.001 |
| **PRKCB** | chr16 | 24230888 | G | A | rs78946887 | UTR3 | - | 0.000 | 0.022 | 0.2184 | 0.000 | 0.005 | 0.0211 | 0.007 |
| **PLCB1** | chr20 | 8720976 | A | C | rs117816042 | intronic | - | 0.000 | 0.033 | 0.1012 | 0.006 | 0.018 | 0.0216 | 0.004 |
| **PLCB4** | chr20 | 9360833 | TA | T | rs11360322 | intronic | - | 0.010 | 0.078 | 0.0268 | 0.005 | 0.016 | 0.0217 | 0.183 |
| SLC41A1 | chr1 | 205764433 | A | C | rs56052552 | intronic | - | 0.010 | 0.059 | 0.1185 | 0.020 | 0.037 | 0.0221 | 0.024 |
| EGFR | chr7 | 55238380 | A | G | rs17336995 | UTR3 | - | 0.000 | 0.022 | 0.2184 | 0.001 | 0.008 | 0.0262 | 0.014 |
| YWHAZ | chr8 | 101966447 | A | G | . | upstream | - | 0.039 | 0.000 | 0.1240 | 0.022 | 0.010 | 0.0335 | - |
| **PLCH1** | chr3 | 155210674 | A | G | . | intronic | - | 0.020 | 0.000 | 0.4993 | 0.006 | 0.000 | 0.0336 | - |
| HDAC4 | chr2 | 239972369 | C | T | rs10174562 | UTR3 | - | 0.196 | 0.033 | 0.0006 | 0.148 | 0.117 | 0.0424 | 0.159 |
| BACE2 | chr21 | 42537986 | C | T | rs28656880 | upstream | - | 0.020 | 0.067 | 0.1500 | 0.048 | 0.070 | 0.0432 | 0.071 |
| CAMK2B | chr7 | 44258803 | C | T | . | UTR3 | - | 0.020 | 0.000 | 0.4993 | 0.007 | 0.001 | 0.0433 | - |
| **PRKCQ** | chr10 | 6624156 | A | G | rs73619607 | ncRNA  _exonic | - | 0.020 | 0.000 | 0.4993 | 0.010 | 0.002 | 0.0463 | 0.008 |
| RELN | chr7 | 103292112 | T | G | rs115734214 | exonic | NS | 0.020 | 0.029 | 1.0000 | 0.010 | 0.021 | 0.0464 | 0.017 |
| GHRL | chr3 | 10331457 | G | T | rs696217 | exonic | NS | 0.078 | 0.167 | 0.0861 | 0.120 | 0.150 | 0.0472 | 0.083 |
| MAPK9 | chr5 | 179720380 | T | G | rs1363513 | intergenic | - | 0.039 | 0.000 | 0.1240 | 0.018 | 0.008 | 0.0500 | 0.082 |

Bold indicates PKC and PLC family genes. Syn refers to synonymous exonic variants and NS refers to non-synonymous exonic variants. cent. refers to centenarians and 1000 Gen. is 1000 Genome Project. p-values are based on Fisher’s exact test.

**Supplementary table 7. Genotyping analysis result of selected 222 variants from the Stage 1 Capture-seq**

- Separate file

**Supplementary Table 8. The list of 217 candidate genes for the Stage 2 Capture-seq**

| ADAM10 | CHUK | HTR3C | NFATC3 | PLCD1 | REL |
| --- | --- | --- | --- | --- | --- |
| ADAM17 | CLSTN1 | HTR3D | NFATC4 | PLCD3 | RELA |
| ADCY1 | CLU | HTR3E | NFKB1 | PLCD4 | RELB |
| ADCY10 | CR1 | HTR4 | NFKB2 | PLCE1 | RPS6KA1 |
| ADCY2 | CREB1 | HTR5A | NFKBIA | PLCG1 | SH2B1 |
| ADCY3 | CREB3 | HTR6 | NFKBIB | PLCG2 | SH2B2 |
| ADCY4 | CREB5 | HTR7 | NOS1AP | PLCH1 | SHC1 |
| ADCY5 | CRK | IKBKB | NRAS | PLCH2 | SHC2 |
| ADCY6 | CRTC1 | IKBKG | NTRK1 | PLCZ1 | SLC41A1 |
| ADCY7 | CRTC2 | IL18 | NTRK2 | PPP2R1A | SNCA |
| ADCY8 | CRTC3 | IL1B | NTRK3 | PPP3CA | SOCS3 |
| ADCY9 | DRD1 | IL1R1 | NUCKS1 | PPP3CB | TFCP2 |
| ADRA1A | DRD1 | IL1R2 | OPRK1 | PPP3CC | TLR2 |
| ADRA1B | DRD2 | IL6 | PDE10A | PPP3R1 | TLR4 |
| ADRA1D | DRD3 | IL6R | PDE11A | PPP3R2 | TNFRSF1A |
| ADRA2A | DRD4 | JUN | PDE1A | PRKACA | TNFRSF1B |
| ADRA2B | DRD5 | KRAS | PDE1B | PRKACB | YWHAZ |
| ADRA2C | EGF | LIPG | PDE1B | PRKACG |  |
| ADRB1 | EGFR | MAP2K1 | PDE1C | PRKAR1A |  |
| ADRB2 | EIF4EBP1 | MAP2K2 | PDE2A | PRKAR1B |  |
| ADRB3 | FOS | MAP3K5 | PDE3A | PRKAR2A |  |
| APOE | GCG | MAPK1 | PDE3B | PRKAR2B |  |
| APP | GLP1R | MAPK10 | PDE4A | PRKCA |  |
| ARAF | GNRH1 | MAPK11 | PDE4B | PRKCB |  |
| ATF2 | GNRH2 | MAPK12 | PDE4C | PRKCD |  |
| ATF4 | GNRHR | MAPK13 | PDE4D | PRKCE |  |
| BACE1 | GRB2 | MAPK14 | PDE5A | PRKCG |  |
| BDNF | GRIK5 | MAPK15 | PDE7A | PRKCH |  |
| BRAF | GSK3B | MAPK3 | PDE7B | PRKCI |  |
| CALM1 | HRAS | MAPK4 | PDE8A | PRKCQ |  |
| CALM2 | HTR1A | MAPK6 | PDE8B | PRKCZ |  |
| CALM3 | HTR1B | MAPK7 | PDPK1 | PRKD1 |  |
| CAMK2A | HTR1D | MAPK8 | PICALM | PRKD2 |  |
| CAMK2B | HTR1E | MAPK9 | PKN1 | PRKD3 |  |
| CAMK2D | HTR1F | MAPT | PKN2 | PSEN1 |  |
| CAMK2G | HTR2A | MTOR | PKN3 | PSEN2 |  |
| CAMKK1 | HTR2B | MYD88 | PLCB1 | PTGS1 |  |
| CAMKK2 | HTR2C | NFAT5 | PLCB2 | PTPN1 |  |
| CH25H | HTR3A | NFATC1 | PLCB3 | PTPN11 |  |
| CHRM5 | HTR3B | NFATC2 | PLCB4 | RAF1 |  |

**Supplementary Table 9. The list of significantly longevity-associated variants (p < 0.05) in 217 candidate genes from the Stage 2 Capture-seq**

- Separate file

**Supplementary Table 10. Characteristics and numbers of variants in 217 candidate genes from the Stage 2 Capture-seq**

| Genomic Region | All variants | % (All variants) | Novel variants | % (Novel variants) | MAF < 0.05 | % (MAF < 0.05) | p < 0.05 | %  (p <0.05) |
| --- | --- | --- | --- | --- | --- | --- | --- | --- |
| downstream | 505 | 2.14 | 262 | 2.17 | 411 | 2.15 | 9 | 1.60 |
| exonic | 2452 | 10.38 | 1122 | 9.28 | 2160 | 11.28 | 51 | 9.04 |
| exonic;splicing | 13 | 0.06 | 4 | 0.03 | 10 | 0.05 | 0 | 0.00 |
| intergenic | 776 | 3.28 | 451 | 3.73 | 614 | 3.21 | 14 | 2.48 |
| intronic | 12039 | 50.96 | 5913 | 48.92 | 9550 | 49.87 | 304 | 53.90 |
| splicing | 35 | 0.15 | 30 | 0.25 | 35 | 0.18 | 1 | 0.18 |
| upstream | 3157 | 13.36 | 1768 | 14.63 | 2527 | 13.20 | 91 | 16.13 |
| UTR3 | 4011 | 16.98 | 2165 | 17.91 | 3321 | 17.34 | 86 | 15.25 |
| UTR5 | 637 | 2.70 | 371 | 3.07 | 520 | 2.72 | 8 | 1.42 |
| Total | 23625 | 100.00 | 12086 | 100.00 | 19148 | 100.00 | 564 | 100.00 |

**Supplementary Table 11. Enrichment analysis to identify sub-pathway genes of PKC and PKC-interacting pathways enriched with longevity-associated genes from the Stage 2 Capture-seq**

| Candidate pathway | Sub-pathway | Total number of genes included in Capture-seq | Number of longevity-associated genes by SKAT (p<0.05) | Enrich-ment  Ratio | Enrich-  ment  p-value |
| --- | --- | --- | --- | --- | --- |
| Receptor/upstream | Serotonin receptor | 17 | 0 | 0 | 1 |
|  | Adrenergic receptor | 9 | 0 | 0 | 1 |
|  | Dopamine receptor | 6 | 0 | 0 | 1 |
|  | Other neuronal receptor | 3 | 0 | 0 | 1 |
|  | EGF receptor signal | 3 | 1 | 0.333 | 0.275 |
|  | Inflammatory signal | 14 | 2 | 0.143 | 0.639 |
|  | Neurotrophin signal | 11 | 0 | 0 | 0.608 |
|  | GnRH signal | 5 | 0 | 0 | 1 |
| PKC signal | PLC | 13 | 2 | 0.154 | 0.627 |
| PKC signal  (PKC family) | Conventional PKC | 3 | 0 | 0 | 1 |
|  | Novel PKC (with PKD) | 7 | 3 | **0.429** | **0.024** |
|  | Atypical PKC (with PKN) | 5 | 1 | 0.200 | 0.417 |
| PKA signal | Phosphodiesterase | 18 | 1 | 0.056 | 1 |
|  | Adenylyl Cyclase | 10 | 1 | 0.100 | 1 |
|  | PKA | 7 | 1 | 0.143 | 0.532 |
| MAPK signal | MAPK | 13 | 2 | 0.154 | 0.627 |
|  | MAPK upstream | 9 | 1 | 0.111 | 1 |
| CaMK signal | CaMK | 6 | 0 | 0 | 1 |
|  | Calmodulin | 3 | 0 | 0 | 1 |
|  | Calcineurin | 5 | 0 | 0 | 1 |
| NF-kB signal | NF-kB complex | 7 | 3 | **0.429** | **0.024** |
|  | IKK | 3 | 0 | 0 | 1 |
| Downstream  factors | NFAT | 5 | 2 | 0.400 | 0.081 |
|  | CREB | 7 | 1 | 0.143 | 0.532 |
|  | CRTC | 3 | 0 | 0 | 1 |
| Other signal | AD pathway | 7 | 0 | 0 | 1 |
|  | mTOR pathway | 4 | 0 | 0 | 1 |
| Neuronal disorder | AD/PD GWAS | 11 | 1 | 0.091 | 1 |
| Lipid | Cholesterol metabolism | 3 | 0 | 0 | 1 |

*Bold indicates significant enrichment of sub-pathways (p < 0.05). Enrichment ratio was determined by dividing sum of number of longevity-associated genes by SKAT (p < 0.05) by sum of total number of genes included in the Stage 2 Capture-seq candidate genes and p-values were based on Fisher’s exact test.

**Supplementary Table 12. Significant IPA canonical pathways (p < 0.05) enriched with significantly longevity-associated genes from the Stage 2 Capture-seq**

| Ingenuity Canonical Pathways | p-value | Genes |
| --- | --- | --- |
| Role of pattern recognition receptors in recognition of bacteria and viruses | 0.0005 | ***PRKCD, PRKCH, PRKD3, RELA, NFKB1,***  *PLCG2, MAPK9, CREB1, IL1B* |
| Production of nitric oxide and reactive oxygen species in macrophages | 0.0027 | ***PRKCD, PRKCH, PRKD3, RELA, NFKB1, NFKBIA,***  *PLCG2, MAPK9, CLU, PPP2R1A* |
| Role of macrophages, fibroblasts and endothelial cells in rheumatoid arthritis | 0.0039 | ***PRKCD, PRKCH, PRKD3, RELA, NFKB1, NFKBIA,***  *PLCG2, PLCB1, MAPK9, CREB1, IL1B, NFATC2, NFATC3, IL6R* |
| Atherosclerosis signaling | 0.0045 | ***PRKCD, PRKCH, PRKD3, RELA, NFKB1****, IL1B, CLU* |
| Phospholipase C signaling | 0.0120 | ***PRKCD, PRKCH, PRKD3, RELA, NFKB1,***  *PLCG2, PLCB1, CREB1, NFATC2, NFATC3, ADCY1* |
| NF-κB activation by viruses | 0.0288 | ***PRKCD, PRKCH, PRKD3, RELA, NFKB1, NFKBIA*** |
| Type II diabetes mellitus signaling | 0.0288 | ***PRKCD, PRKCH, PRKD3, RELA, NFKB1, NFKBIA****, MAPK9* |
| Protein kinase A signaling | 0.0389 | ***PRKCD, PRKCH, PRKD3, RELA, NFKB1, NFKBIA,*** *PLCG2,*  *PLCB1, CREB1, NFATC2, NFATC3, ADCY1, PRKCAR2A, PDE4B* |
| Huntington's disease signaling | 0.0467 | ***PRKCD, PRKCH, PRKD3,*** *PLCB1, MAPK9, CREB1, EGFR* |

*Bold indicates PKC family and NF-κB complex genes.

**SUPLLEMENT EXPERIMENTAL PROCEDURES**

**Library preparation, target enrichment and sequencing for the Stage 1 Capture-seq**

Genomic DNA from the 51 centenarians and 51 controls from the AJ population were used to generate libraries for Illumina next-generation sequencing. Six centenarian samples were prepared separately as was done in a previous report ([Han et al., 2013](#_ENREF_1)). For 96 samples, including 51 controls and the 45 centenarians, 12-multiplex pre-pooled target capture sequencing was performed for 8 lanes of sequencing by Illumina HiSeq2000.

Each library was indexed with a unique barcode, which allowed for target capture, enrichment of pooled libraries, and multiplex sequencing. The library preparation was performed according to the Illumina TruSeq DNA sample preparation v2 low-throughput (LT) protocol with some modifications. The 12-plex pre-pooled target capture was performed according to the Agilent SureSelect Target Enrichment Protocol with some modification. Target-enriched libraries were sent to Axeq Technologies and sequenced paired-end on the Illumina HiSeq2000 with cluster kit v3 according to the manufacturer’s protocol. Reads generated were 101 bp in length.

**Data analysis for the Stage 1 Capture-seq of individually indexed target capture sequencing**

Sequencing reads were aligned to the human genome, revision hg19, using BWA, version 0.5.9. Following alignment, we utilized Picard (http://picard.sourceforge.net) to detect potential PCR duplicates and to calculate on-target statistics. Subsequent processing to identify variants was performed using the Genome Analysis Toolkit (GATK). The SNP databases used were the 1000 Genomes Project, dbSNP build 135, and HapMap3 r3. Functional annotations, including SIFT and PolyPhen-2 scores, were provided by ANNOVAR.

**Library preparation, target enrichment and sequencing for the Stage 2 Capture-seq**

We used genomic DNA from 450 centenarians and 550 controls (500 controls for the 2nd Stage 2 Capture-seq) of AJ population and generated libraries for Illumina next-generation sequencing. Twenty-five samples in the same group were pooled for equimolar concentration to make up 1ug of DNA in total for one pool. For the 18 pools of centenarians and 22 (20 for 2nd Pool-seq) pools of controls, multiplex pre-pooled target capture sequencing was performed for sequencing by Illumina HiSeq2000.

Each pooled library was indexed with a unique barcode, which allowed identification of each pool for target capture, enrichment of pooled libraries, and multiplex sequencing. The library preparation was performed according to the Illumina TruSeq DNA sample preparation v2 low-throughput (LT) protocol with some modifications. The multiplex pre-pooled target capture was performed according to the Nimblegen SeqCap EZ Choice library target capture protocol, with some modifications. Target-enriched libraries were sent to Axeq Technologies and sequenced paired-end on the Illumina HiSeq2000 with cluster kit v3 according to the manufacturer’s protocol. Reads generated were 101 bp in length.

**Data analysis for the Stage 2 Capture-seq**

The BWA alignment software, version 0.7.5a was used to align the sequence data to the hg19 human reference genome (GRCh37 assembly, February 2009). The hg19 human reference genome was downloaded from the UCSC Genome Browser. Potential PCR duplicates were removed with the rmdup routine of the samtools software, version 0.1.18. The Picard tools, version 1.81, were used to collect statistics about the read coverage in each pool. Genetic variants were called using the software CRISP, which was specifically developed to call variants in pooled DNA sequence data. Functional annotations were assigned to the variants using the SG-ADVISER annotation pipeline software (http://genomics.scripps.edu/ADVISER/). This annotation pipeline generates a large compilation of predicted effects of the variants to functional features.

**REFERENCES**

Han, J., Ryu, S., Moskowitz, D. M., Rothenberg, D., Leahy, D. J., Atzmon, G., . . . Suh, Y. (2013). Discovery of novel non-synonymous SNP variants in 988 candidate genes from 6 centenarians by target capture and next-generation sequencing. *Mech Ageing Dev*. doi: 10.1016/j.mad.2013.01.005
